# Mapping the intellectual structure and emerging trends on nanomaterials in colorectal cancer: a bibliometric analysis from 2003 to 2024

**DOI:** 10.3389/fonc.2024.1514581

**Published:** 2025-01-08

**Authors:** Man Lu, Yi Liu, Jin Zhu, Jiarong Shang, Lu Bai, Zhichao Jin, Wenting Li, Yue Hu, Xia Zheng, Jun Qian

**Affiliations:** ^1^ No. 1 Clinical Medical College, Nanjing University of Chinese Medicine, Nanjing, Jiangsu, China; ^2^ Department of Oncology, Affiliated Hospital of Nanjing University of Chinese Medicine, Nanjing, China

**Keywords:** colorectal cancer, nanomaterial, bibliometric analysis, visualization, R-bibliometrix, research trends

## Abstract

**Background:**

Colorectal cancer (CRC) is one of thes most prevalent malignant tumors worldwide. Current therapeutic strategies for CRC have limitations, while nanomaterials show significant potential for diagnosing and treating CRC. This study utilizes bibliometric analysis to evaluate the current status and trends in this field.

**Methods:**

Research on nanomaterials in CRC from 2003 to 2024 was retrieved from the Web of Science Core Collection (WOSCC). Tools such as CiteSpace, VOSviewer, RStudio, GraphPad Prism, and Excel were used to analyze trends and hotspots, covering publication trends, countries, institutions, authors, journals, co-citation analysis, and keywords. Visual maps were created to forecast future developments.

**Results:**

The analysis includes 3,683 publications by 17,261 authors from 3,721 institutions across 100 countries/regions, published in 840 journals. Global publications have steadily increased, particularly since 2018. China leads in publication volume and citations, with six of the top ten research institutions and seven of the ten most cited authors, while the United States excels in citation impact and academic centrality. Both countries currently dominate the field, underscoring the urgent need for enhanced international collaboration. Ramezani M and Abnous K lead in publication volume and H-index, while Siegel RL is highly cited. The International Journal of Nanomedicine has the highest publication volume, while the Journal of Controlled Release is the most cited. In addition to “colorectal cancer” and “nanoparticles,” the most common keyword is “drug delivery.” Emerging research areas such as “metal-organic frameworks (MOFs)” and “green synthesis” are gaining attention as leading hotspots.

**Conclusion:**

This study offers an in-depth analysis of the application of nanomaterials in CRC, promoting interdisciplinary collaboration and advancing scientific progress in this field.

## 1 Introduction

CRC is one of the most common malignant tumors worldwide, with its incidence rate increasing while the rate of decline in mortality has slowed in recent years (1, 2). By 2023, approximately 153,020 individuals were diagnosed with CRC, and 52,550 succumbed to this disease, imposing significant economic burdens on society and affected families (3, 4).

The treatment methods for CRC include local therapies and systemic therapies. Local therapies primarily consist of surgery, radiotherapy, ablation, and embolization. For early-stage CRC, surgery is considered the optimal treatment choice. Systemic therapies include chemotherapy, targeted therapy, and immunotherapy. Commonly used chemotherapeutic agents, such as 5-fluorouracil (5-FU) and oxaliplatin, can effectively destroy cancer cells, but they may also cause adverse reactions such as nausea and vomiting. Despite numerous treatment strategies employed for CRC, each technique has inherent limitations that hinder the achievement of expected therapeutic effects (5). For example, anticancer drugs face various issues, including nonspecific distribution (6), rapid clearance (7), drug resistance (8), and toxic reactions (9), all of which can diminish treatment efficacy. Therefore, searching for new therapeutic strategies has become a crucial direction in current research, and the rapid development of nanomaterials offers new hope for CRC treatment (10).

Nanomaterials, due to their unique properties such as size effects, surface effects, quantum effects, multifunctionality, and excellent biocompatibility, hold great promise for the diagnosis and treatment of CRC. These attributes enhance diagnostic accuracy and therapeutic efficacy. In diagnosis, nanomaterials improve imaging techniques (e.g., CT, MRI, and fluorescence imaging), boosting the sensitivity and precision of early tumor detection (11). By binding to cancer biomarkers, they enable targeted identification of tumor cells and early-stage lesions. In therapy, their multifunctionality makes them ideal for drug delivery and targeted treatment. Nanomaterials can be surface-functionalized to target specific tumor cell receptors, increasing drug concentration at the tumor site, enhancing treatment efficacy, and minimizing off-target effects (12). Additionally, they can be engineered to respond to tumor microenvironment changes, enabling controlled drug release and further improving therapeutic precision. In particular, the combination of nanomaterials with other modalities, such as chemotherapy, radiotherapy, and immunotherapy (13), enhances synergistic effects, overcomes resistance, and enables a more individualized treatment approach for CRC.

We conducted a visual analysis of the research distribution of nanomaterials across various types of cancer, revealing a considerable body of literature focused on the application of nanomaterials in the diagnosis and treatment of CRC (**Supplementary Figure S1**). However, there is currently a lack of systematic bibliometric analysis in this area. The study aims to comprehensively utilize bibliometric methods to examine the research history and current status of nanomaterials in CRC over the past two decades. This will assist the academic community in gaining a more thorough understanding of the research hotspots and future directions in this domain.

## 2 Materials and methods

### 2.1 Data source

Our data are sourced from the WOSCC, which is a comprehensive, multidisciplinary journal citation database widely recognized for its extensive coverage. Each article in this database contains metadata such as publication year, country and region, authorship details, institutional affiliations, document type, research field, journal title, citation counts, and references (14). Researchers generally regard such databases as highly suitable for conducting bibliometric analysis.

### 2.2 Search strategy

**Figure 1:**
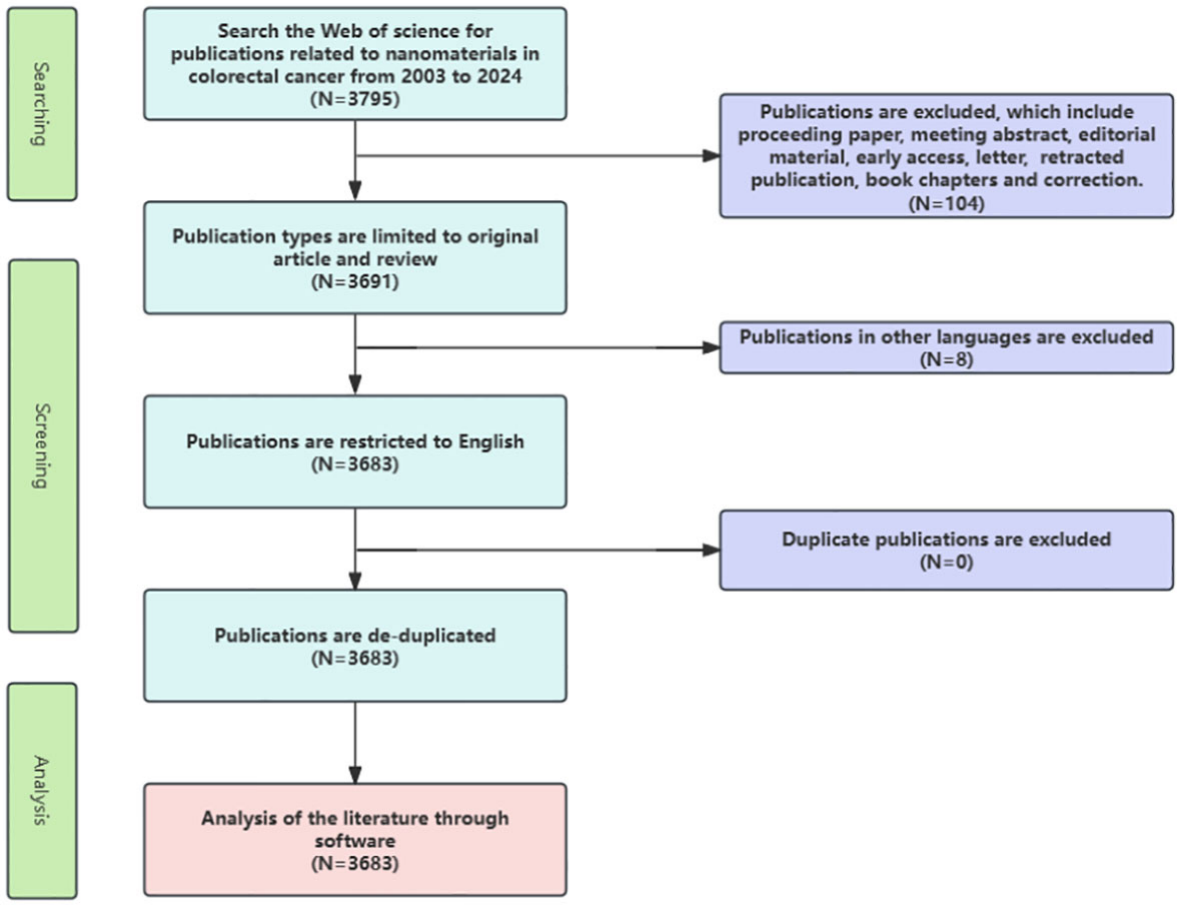
Flowchart of this study.

We systematically searched and compiled relevant publications concerning nanomaterials in CRC from January 1, 2003, to July 10, 2024. Our study’s inclusion criteria included (1) Full-text publications relevant to nanomaterials in CRC, (2) Articles (research reports that are considered to be citable new and original works), and reviews (detailed, important surveys of published research) written in English. Exclusion criteria comprised: (1) Publications unrelated to nanomaterials in CRC; (2) Types such as proceeding papers (full papers presented at symposia or conferences across various disciplines), meeting abstracts (summaries or extended summaries of reports presented at workshops or conferences), editorial materials (essays expressing the views of individuals, groups, or organizations), early access (papers published electronically by journals before being assigned a volume and issue number), letters (brief correspondence between readers and journal editors on past published material), retracted publications (papers withdrawn by authors, institutions, editors, or publishers), book chapters (monographs or publications on specific topics found within books), and corrections(revisions made to correct errors identified post-publication). The search was conducted on a single day to maintain uniformity in data acquisition. Data were exported in the format of “full records and cited references” as plain text, as illustrated in **Figure 1**, depicting the screening process.

The following is the search formula: TS = (nanodot* OR nanoparticle* OR nanomaterial* OR nanotube* OR nanosheet* OR “quantum dot*” OR nanofiber* OR nanosphere* OR nanorod* OR nanowire* OR nanocrystal* OR nanocomposite* OR nanodevice* OR nanocluster* OR nanotechn* OR nanocarrier* OR nanowire* OR nanoliposome* OR nanoemulsion* OR nanocrystal* OR nanoconjugate* OR nanogels* OR nanodiamond* OR nanoporou* OR nanosilver* OR nanopore* OR nanomicell* OR nano size* OR nanomedicine* OR nanofibrou*)AND (((((((((((((((TS=(Colorectal Neoplasms)) OR TS=(Colorectal Neoplasm)) OR TS=(Neoplasm, Colorectal)) OR TS=(Neoplasms, Colorectal)) OR TS=(Colorectal Tumors)) OR TS=(Colorectal Tumor)) OR TS=(Tumor, Colorectal)) OR TS=(Tumors, Colorectal)) OR TS=(Colorectal Cancer)) OR TS=(Cancer, Colorectal)) OR TS=(Cancers, Colorectal)) OR TS=(Colorectal Cancers)) OR TS=(Colorectal Carcinoma)) OR TS=(Carcinoma, Colorectal)) OR TS=(Carcinomas, Colorectal)) OR TS=(Colorectal Carcinomas).

We conducted bibliometric analysis on the collected publications for comprehensive data analysis and visualization. This involved examining multiple aspects, including countries and regions of publications, affiliated institutions, authors, journals, and their impact factor (IF), citation counts, references, and keywords. To ensure rigor, two researchers independently analyzed the data and cross-verified the results to maintain the study’s accuracy and reproducibility.

### 2.3 Data analysis

To quantify the data, we conducted a quantitative analysis focusing on the number of annual publications and their trends. This analysis included ranking the top ten countries/regions, institutions, authors, and journals based on publication volume. Additionally, we performed a qualitative study of the relationships between authors and co-cited authors, journals and co-cited journals, and co-cited references. Furthermore, we comprehensively analyzed the collaborative relationships among different countries/regions, institutions, and authors and the research trends observed over the past twenty years.

### 2.4 Bibliometric and visualized analysis

We utilized CiteSpace (version 6.2.R4) for co-citation, co-word, and keyword analysis (15), helping researchers identify research hotspots and frontiers and visualize current research trends (16). We also employed VOSviewer (version 1.6.18) to create visual maps of different authors, journals, and keywords to analyze the literature clustering (17, 18). Moreover, we used RStudio (bibliometrix package) to explore the current research structure and future research trends and identify shifts in research hotspots (19). Additionally, GraphPad Prism (version 8.0.2) and Microsoft Excel 2010 were used for quantitative visualization of specific data. These software tools enabled us to extract critical insights from many publications, providing valuable perspectives for our research.

## 3 Results

### 3.1 Global trend of publications

**Figure 2:**
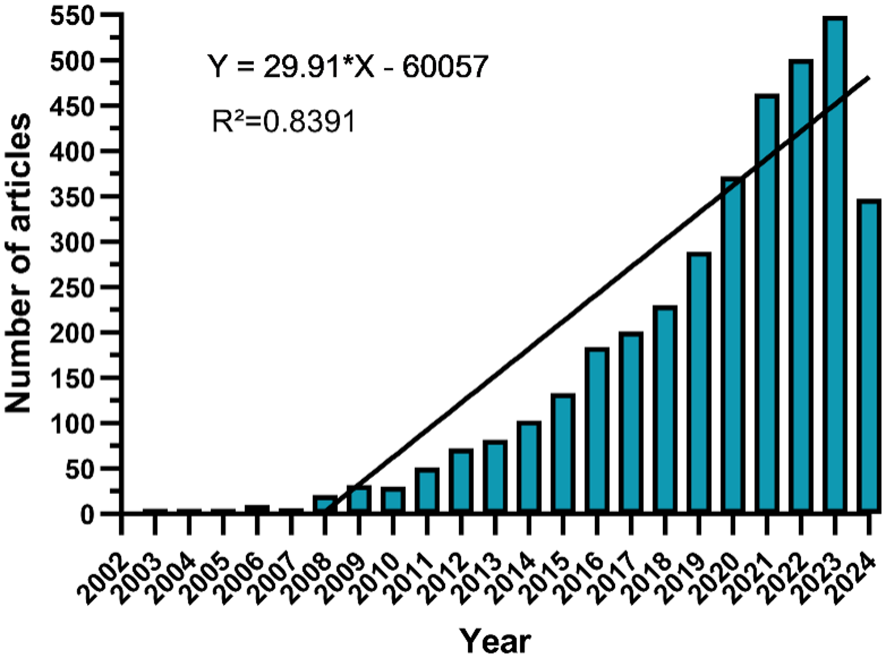
Annual publications and trends.

This study retrieved 3,683 publications related to nanomaterials in CRC from the WOSCC database, comprising 3,083 articles (83.7%) and 600 review articles (16.3%). The annual publications and their trends in this field are shown in **Figure 2**. The number of publications has notably increased from just 1 in 2003 to 549 in 2023. We categorized the overall trend into three stages: initially slow growth from 2003 to 2007, with annual publications numbering less than 10, indicating the field was in its infancy; a steady increase from 2008 to 2017; and rapid expansion post-2018, reaching a peak in 2023. Based on this trend, the annual publication volume is potentially projected to achieve a new high by the end of 2024.

### 3.2 Analysis of countries/regions

**Figure 3:**
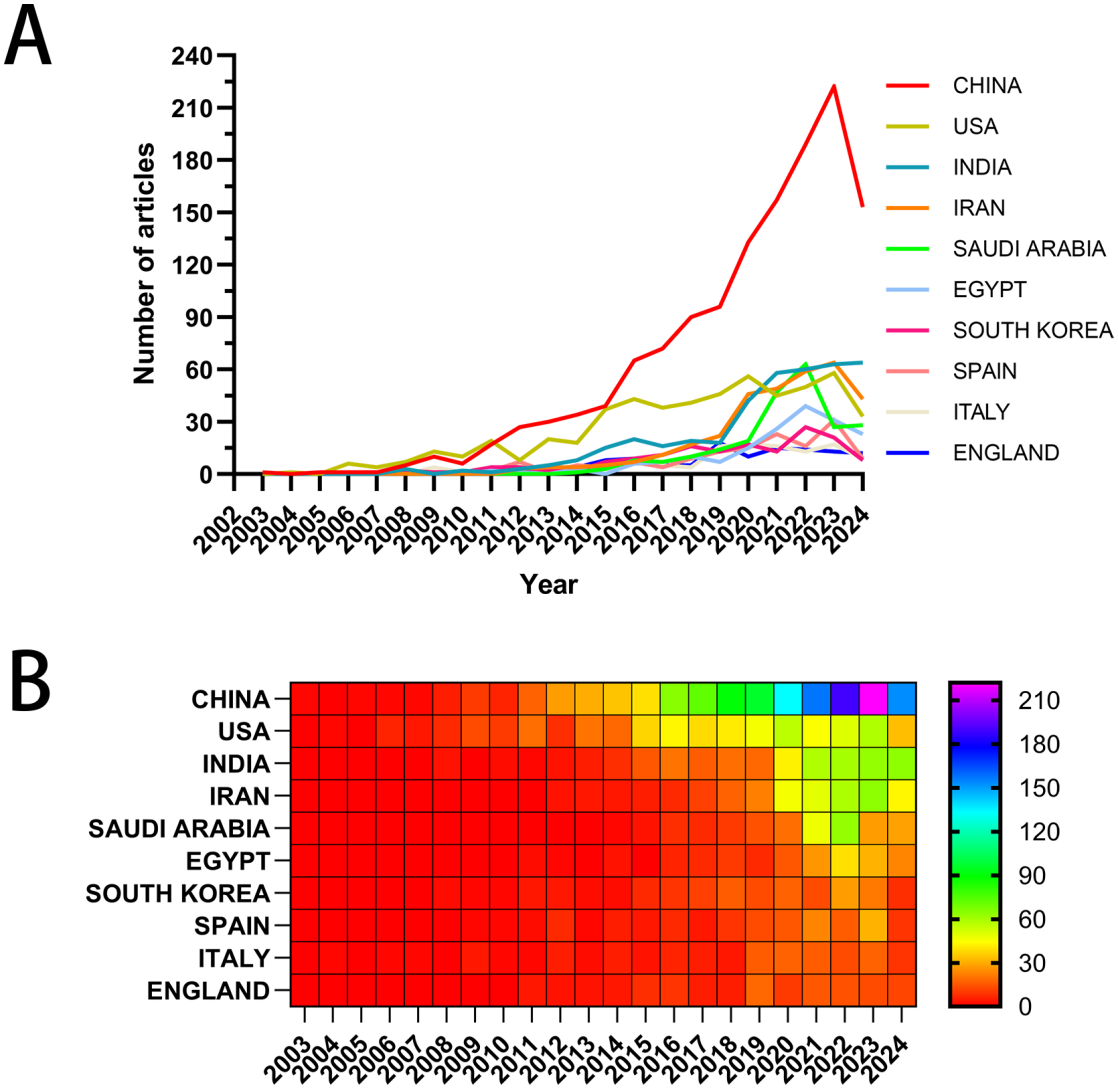
Visual mapping of the origin of the countries of publications. **(A)** Line graph of national publications; **(B)** Heat map of national publications.

In **Figure 3A**, the x-axis represents the years, and the y-axis denotes the publication counts, with different colored lines indicating the publication trends for each country. In **Figure 3B**, the x-axis represents the years, and the y-axis corresponds to the countries, with publication volumes depicted through a color gradient from red to purple. Both the line graph and the heat map display the annual publication counts and trends for the top ten high-producing countries/regions. **Supplementary Table S1** offers detailed information regarding publication volume and citation counts for these countries/regions. China ranks first with 1,349 papers, comprising 36.63% of the total, followed by the United States with 556 papers, India with 397 papers, Iran with 334 papers, and Saudi Arabia with 227 papers. The total citation counts for Chinese papers reach 39,241, far exceeding all other countries/regions, with an average citation count of 29.09 per paper, ranking third among all nations. The United States ranks second with 556 publications and 25,888 total citations. However, it boasts an impressive average of 46.56 citations per paper and a centrality score of 0.31, placing it first in these two metrics. The inter-country collaboration network is shown in **Supplementary Figure S2**. China and the United States have the highest number of publications and the closest collaborative relationship. China, with its prolific paper output, collaborates closely with India, France, and the United Kingdom. In contrast, the United States exhibits robust partnerships with Iran, Saudi Arabia, and Egypt.

### 3.3 Analysis of institutions

**Figure 4:**
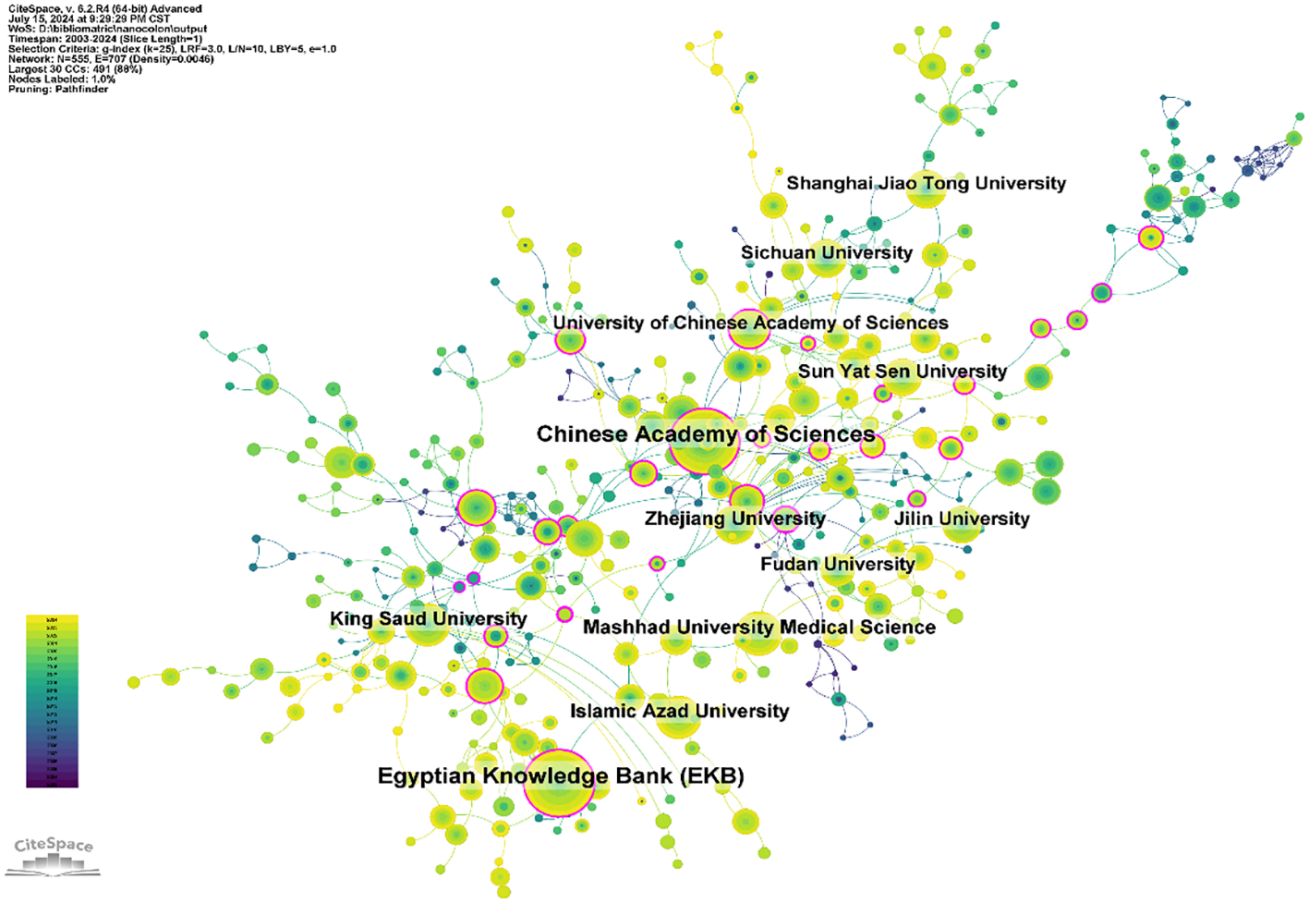
Networks of institutional cooperation.

A total of 3,721 institutions have systematically published papers on nanomaterials in CRC. The top ten institutions for paper publications are listed in **Supplementary Table S2**, with six located in China, two in Iran, one in Egypt, and one in Saudi Arabia. The Chinese Academy of Sciences and the Egyptian Knowledge Bank have each published 159 papers, ranking first in publication volume. The total citation counts of the Chinese Academy of Sciences have reached 6,310, with an average of 39.69 citations per paper, surpassing that of other institutions. Using CiteSpace software, we further analyzed the cooperation networks between institutions, as shown in **Figure 4**. The Chinese Academy of Sciences exhibits extensive collaboration with domestic universities and institutions such as Zhejiang University, Fudan University, Jilin University, and Sichuan University. Similarly, Mashhad University of Medical Sciences establishes closer cooperation with domestic institutions in Iran, such as Islamic Azad University.

### 3.4 Analysis of authors and co-cited authors

In this study, 17,261 authors have contributed to publications on applying nanomaterials in CRC. **Table 1** lists the ten authors with the highest publication volume and the ten authors with the most co-citations. Mangues R, Ramezani M, Vazquez E, and Villaverde A. are the authors of most of the research papers (21 papers). Ramezani M and Abnous K from Iran boast impressive H-index values of 65 and 64, respectively, indicating their significant academic reputation and influence. CiteSpace software was employed to visualize author collaborations in **Supplementary Figure S3A**. Authors from Spain, like Mangues R, Villaverde A, and Unzueta U, form a red cluster, while authors from Iran, such as Ramezani M and Vazquez E, form a green cluster. The substantial dispersion between clusters suggests that authors in this field collaborate more frequently with others from the same nationality. Additionally, we visually analyzed the collaboration network among the 116 authors who were co-cited more than 50 times in **Supplementary Figure S3B**. The most prominent nodes in the figure correspond to the most co-cited authors, including Siegel RL (393 citations), Jemal A (355 citations), and Zhang Y (299 citations). Combining the results from **Table 1**, we observe that among the ten authors with the most co-citations, seven are from China, two from the United States, and one from Iran.

**Table 1:** Top 10 productive authors and co-cited authors producing studies related to nanomaterials in CRC.

Rank	Author	Country	Count	H-index	Rank	Co-cited author	Country	Citation
1	Mangues R	SPAIN	21	33	1	Siegel RL	USA	393
2	Ramezani M	IRAN	21	65	2	Jemal A	USA	355
3	Vazquez E	SPAIN	21	38	3	Zhang Y	CHINA	299
4	Villaverde A	SPAIN	21	50	4	Wang Y	CHINA	269
5	Unzueta U	SPAIN	20	27	5	Liu Y	CHINA	238
6	Abnous K	IRAN	19	64	6	Li Y	CHINA	223
7	Shieh MJ	CHINA	19	36	7	Li J	CHINA	210
8	Taghdisi SM	IRAN	18	58	8	Maeda H	IRAN	203
9	Huang L	USA	17	36	9	Chen Y	CHINA	202
10	Alibolandi M	IRAN	16	47	10	Wang J	CHINA	201

### 3.5 Analysis of journals and co-cited journals

In this study, all publications are distributed across 840 journals and 13,351 co-cited journals. **Table 2** summarizes the top ten journals and top ten co-cited journals. It provides a comprehensive assessment of their influence based on publication volume, citation count, IF, and Journal Citation Reports (JCR) categories. All listed journals belong to the Q1 category, with the ACS Nano having the highest IF of 15.8. The density map of journal publications is shown in **Supplementary Figure S4A**. By combining the information from **Table 2**, we can determine that the International Journal of Nanomedicine (100 publications) has the highest output in this field, followed by the International Journal of Pharmaceutics (74 publications) and Pharmaceutics (67 publications). Additionally, the co-citation frequency reflects whether a journal has significantly impacted a research field. We performed a network map of co-cited journals, as illustrated in **Supplementary Figure S4B**. The Journal of Controlled Release has the highest co-citation frequency (1728 citations), followed by Biomaterials (1670 citations) and Cancer Research (1461 citations).

**Table 2:** Top 10 productive journals and co-cited journals related to nanomaterials in CRC.

Rank	Journal	Output	IF(2023)	JCR	Co-cited Journal	Citation	IF(2023)	JCR
1	International Journal of Nanomedicine	100	6.6	Q1	Journal of Controlled Release	1728	10.5	Q1
2	International Journal of Pharmaceutics	74	5.3	Q1	Biomaterials	1670	12.8	Q1
3	Pharmaceutics	67	4.9	Q1	Cancer Research	1461	12.5	Q1
4	Journal of Controlled Release	66	10.5	Q1	International Journal of Nanomedicine	1442	6.6	Q1
5	International Journal of Molecular Sciences	60	4.9	Q1	Acs Nano	1366	15.8	Q1
6	ACS Nano	54	15.8	Q1	Advanced Drug Delivery Reviews	1263	15.2	Q1
7	Biomaterials	54	12.8	Q1	International Journal of Pharmaceutics	1262	5.3	Q1
8	Journal of Drug Delivery Science And Technology	53	4.5	Q1	Scientific Reports	1254	3.8	Q1
9	International Journal of Biological Macromolecules	52	7.7	Q1	Plos One	1132	2.9	Q1
10	Scientific Reports	47	3.8	Q1	Proceedings of The National Academy of Sciences of The USA	1126	9.4	Q1

**Figure 5:**
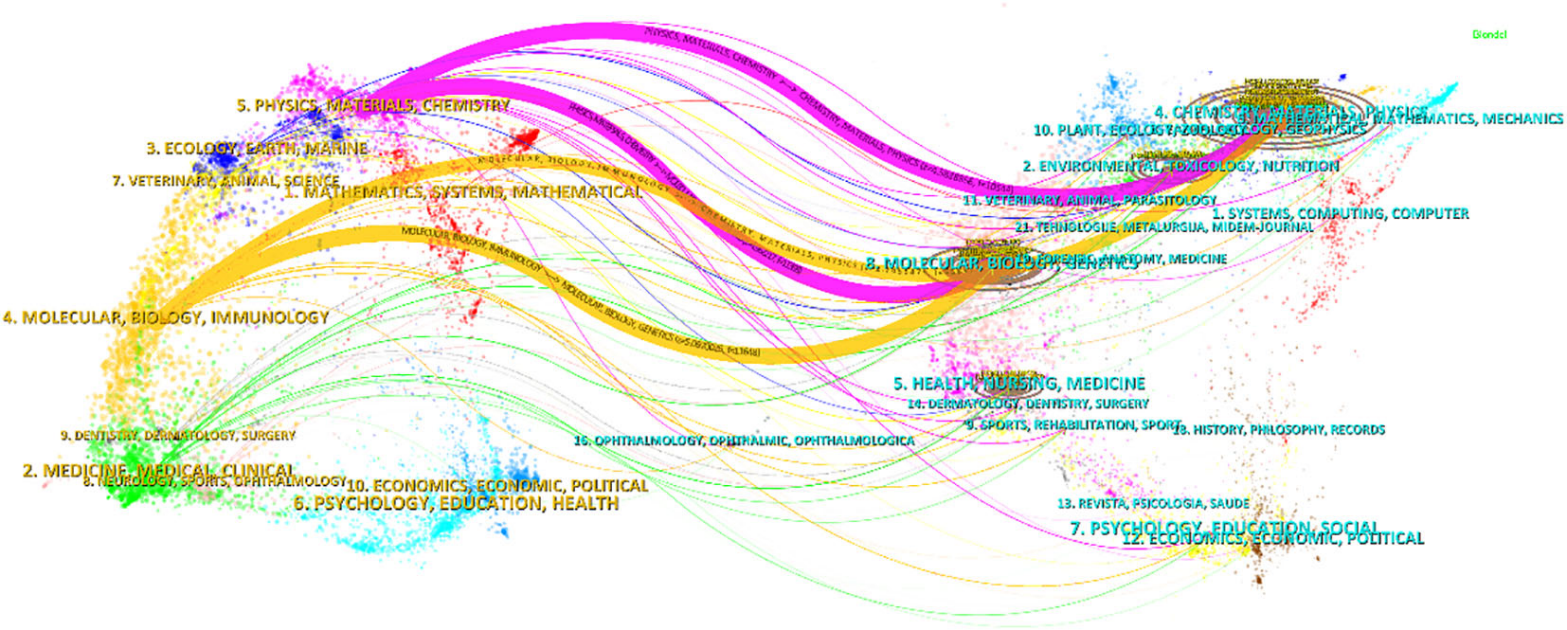
Journal dual-map produced by Citespace showing the citation and cited relationships between journals.

The citation and co-citation relationships between the journals are displayed using an overlapping dual graph (**Figure 5**). Colorful trajectories represent citation connections, with the left side indicating the citing journals and the right side representing the cited journals. Based on the results, we identified four major colored citation pathways, where publications from “Chemistry/Materials/Physics” journals and “Molecular/Biology/Genetics” journals are frequently cited by publications from “Physics/Materials/Chemistry” journals and “Molecular/Biology/Immunology” journals.

### 3.6 Analysis of co-cited references and reference burst

**Supplementary Table S3** presents the top ten highly co-citation references associated with nanomaterials in CRC research. We utilized CiteSpace software to visually analyze these references from 2003 to 2024, segmented by yearly time slices, resulting in a network comprising 1239 nodes and 4658 links (**Supplementary Figure S5A**). The vast majority of these references are published in high-impact Q1 journals. The three most cited papers are as follows: Siegel RL et al., 2021, CA-A Cancer Journal for Clinicians (20); Dekker E et al., 2019, The Lancet (21); and Xie YH et al., 2020, Signal Transduction and Targeted Therapy (22). Notably, Siegel RL and colleagues from the United States have made significant contributions, with their widely cited article providing a comprehensive analysis of current cancer statistics, emphasizing concerns about the deceleration in the decline of CRC mortality rates. Among these ten influential papers, four focus on epidemiological studies of CRC, three provide comprehensive treatment reviews, and the remaining three examine advancements and future directions in cancer nanomedicine. Overall, all these co-cited references represent substantial research contributions to the field.

Additionally, we conducted a cluster analysis on co-cited references (**Supplementary Figure S5B**) and plotted a peak map of co-citation clusters over time (**Supplementary Figure S5C**). Each peak represents a group of closely related literature that revolves around a common theme or research direction. The color of each peak signifies different clusters, while the peak’s size usually indicates the reference citation frequency. By analyzing the changes in clustering over various periods, trends in the development of the research field can be identified. From the figures, it can be seen that early research hotspots include enzyme therapy (cluster 7), targeted contrast agents (cluster 15), and tumor markers (cluster 16). The mid-term research hotspots encompass endoscopic imaging agents (cluster 6), intracellular fate (cluster 8), curcumin (cluster 9), SN38 (cluster 10), *in vivo* delivery (cluster 11), intraperitoneal chemotherapy (cluster 13), microfluidics (cluster 14), poly (cluster 17), indigo carmine (cluster 18), photodynamic therapy (PDT) (cluster 19), and pharmacokinetics (cluster 20). Currently, the popular research directions in this field include colorectal cancer (cluster 0), elderly colorectal cancer patients (cluster 1), extracellular vesicles (cluster 2), polymeric micelles (cluster 3), photothermal therapy (cluster 4), self-assembling (cluster 5), cytotoxicity (cluster 12), human colorectal cancer (cluster 21), and porphyrin (cluster 22).

**Figure 6:**
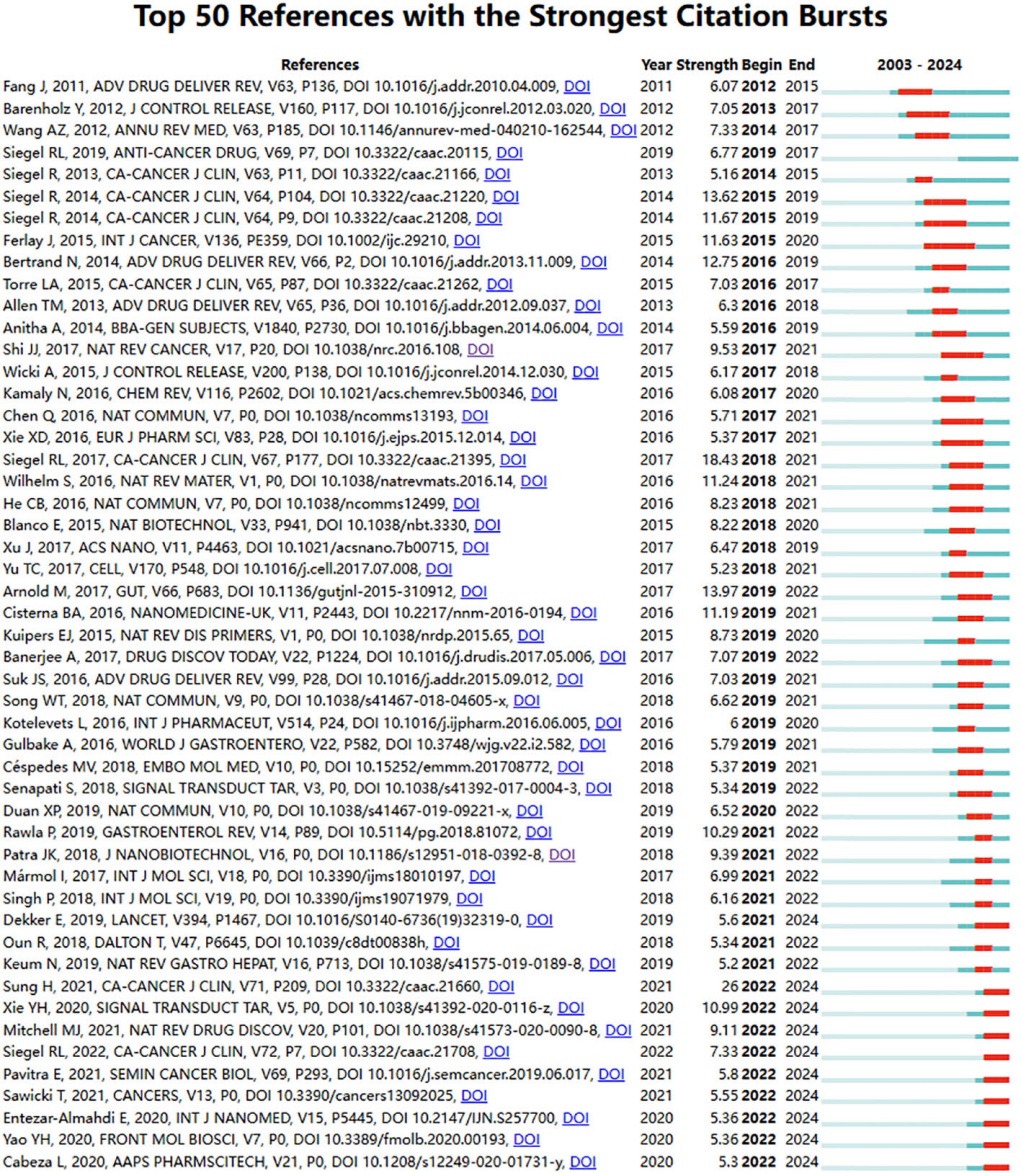
Bursting map of cited references.

We also listed the top fifty references with the strongest citation bursts in nanomaterials in CRC, as shown in **Figure 6**. Among the top ten, six papers focus on overall cancer epidemiology and CRC epidemiology. Excluding these, the three most intensely citation burst references relevant to the topic are:” Cancer nanotechnology: The impact of passive and active targeting in the era of modern cancer biology”(Strength:12.75; Publication Year: 2014) (23), “Analysis of nanoparticle delivery to tumors”(Strength: 11.24; Publication Year: 2016) (24) and “Targeted Nanoparticles for Colorectal Cancer” (Strength:11.19; Publication Year: 2016) (25). Notably, 48 references (accounting for 96%) have a citation burst period spanning from 2014 to 2024, indicating that these references have been frequently cited over the past decade. Additionally, ten references (accounting for 20%; publication years: 2019 to 2021) are experiencing a citation burst. All these mean that nanomaterials in CRC may continue to receive attention in the future.

### 3.7 Analysis of keywords and keywords burst

**Figure 7:**
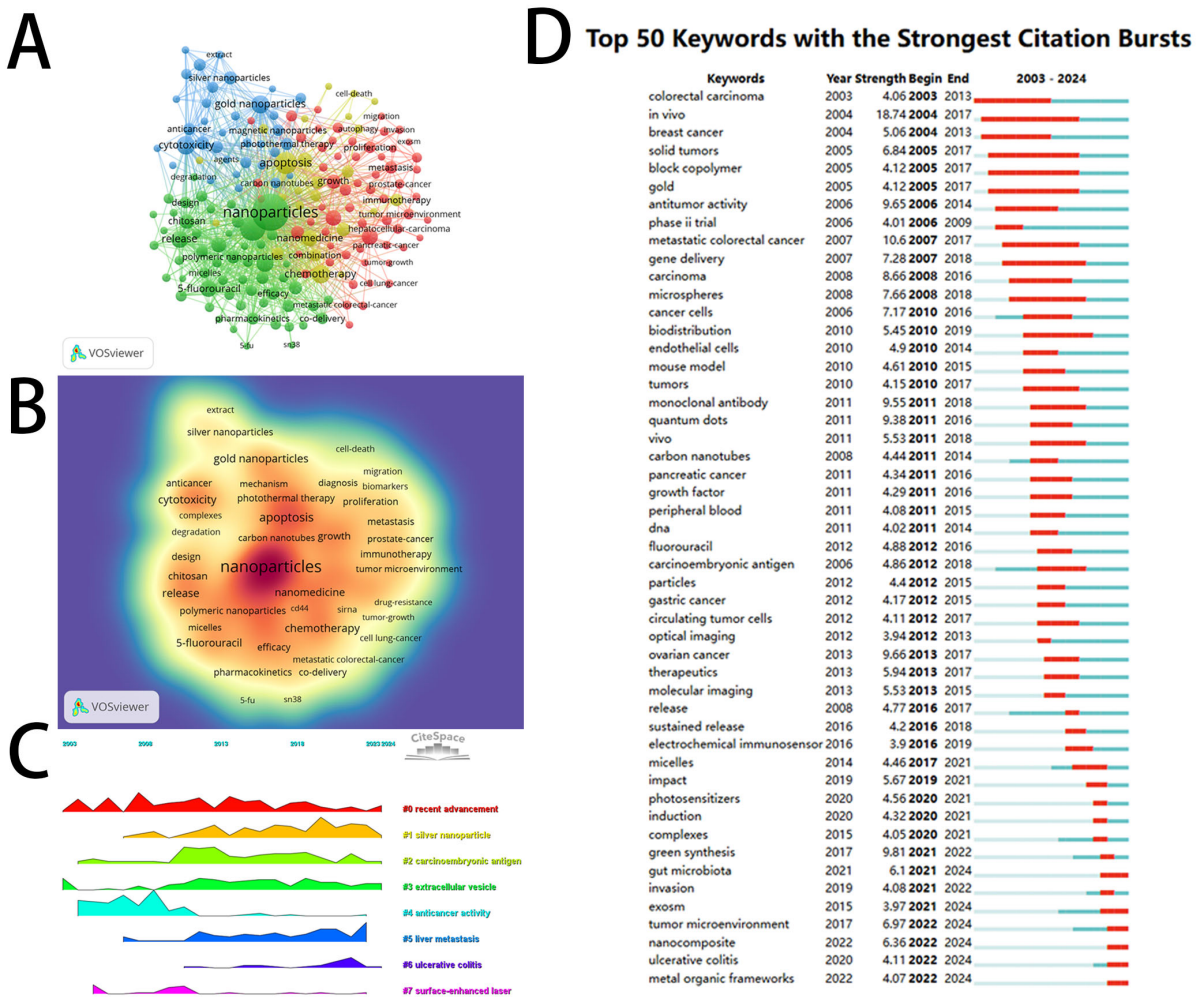
Visual mapping of the keywords. **(A)** Networks of high-frequency keywords; **(B)** Density map of high-frequency keywords; **(C)** Peak map of clustering of topics formed by high-frequency keywords over time; **(D)** Bursting map of high-frequency keywords.

**Table 3** presents the top 20 keywords by frequency, with the most prevalent keyword being “nanoparticles” (1139 times), followed by “drug-delivery” (502 times), “apoptosis” (374 times), and “cytotoxicity” (253 times). Using VOSviewer software, we constructed a visualization network diagram (**Figure 7A**) and a visual density map (**Figure 7B**) of high-frequency keywords after removing irrelevant keywords. The results reveal four distinct clusters represented by different colors: the red cluster contains 61 keywords, primarily focused on the applications of nanomaterials in the diagnosis and treatment of CRC and their impact on the biological behavior of CRC cells, including “diagnosis,” “biomarkers,” “metastasis,” “growth,” “stem cells,” and “exosome.” The green cluster includes 56 keywords, mainly related to nano-drug delivery systems and common nanocarriers, such as “nanoparticle,” “drug-delivery,” “oral delivery,” “bioavailability,” “antitumor activity,” “liposomes,” “polymeric nanoparticles (PNPs),” “microspheres,” and “curcumin.” The blue cluster comprises 34 keywords, primarily addressing the applications of various types of nanomaterials and their cytotoxicity issues, as well as the application of green synthesis methods in their preparation, including “gold nanoparticles (AuNPs),” “silver nanoparticles (AgNPs),” “nanocomposite,” “graphene oxide,” “magnetic nanoparticles (NPs)” “cytotoxicity,” and “green synthesis.” The yellow cluster consists of 26 keywords, mainly focusing on the mechanisms of resistance in CRC treatment and various strategies for overcoming resistance with nanomaterials, including “resistance,” “chemotherapy,” “ferroptosis,” “microenvironment,” and “gut microbiota.”

**Table 3:** Top 20 high-frequency keywords related to nanomaterials in CRC.

Rank	Keyword	Counts	Rank	Keyword	Counts
1	nanoparticles	1139	11	breast-cancer	186
2	drug-delivery	502	12	*in-vivo*	177
3	apoptosis	374	13	Curcumin	159
4	cytotoxicity	253	14	Chitosan	152
5	chemotherapy	252	15	photodynamic therapy	145
6	gold nanoparticles	246	16	Growth	144
7	release	216	17	Resistance	130
8	doxorubicin	209	18	Toxicity	124
9	5-fluorouracil	202	19	targeted delivery	120
10	nanomedicine	188	20	silver nanoparticles	119

CiteSpace software was also employed to generate a peak map illustrating the temporal evolution of high-frequency keywords’ clusters (**Figure 7C**), thereby visually demonstrating the shifts in research hotspots. The peak of “recent advancement” (cluster 0) is relatively broad, indicating sustained attention over the past two decades. “Anticancer activity” (cluster 4) and “surface-enhanced laser” (cluster 7) were research hotspots before 2010, whereas post-2010, the popularity of “silver nanoparticle” (cluster 1), “carcinoembryonic antigen” (cluster 2), “extracellular vesicle” (cluster 3), and “liver metastasis” (cluster 5) has gradually increased and continues to this day. “Ulcerative colitis” (cluster 6) experienced a short-term peak in 2022, after which its popularity has steadily declined.

Among the top 50 keywords with the strongest burst strength in this field (**Figure 7D**), we found that the three keywords with the highest burst intensity are: “*in vivo*” (intensity: 18.74; occurrence year: 2004), “metastatic colorectal cancer” (intensity: 10.6; occurrence year: 2007), and “green synthesis” (intensity: 9.81; occurrence year: 2017). Furthermore, it is noteworthy that six keywords from the top 50 are currently in a state of citation burst, including “metal-organic frameworks,” “ulcerative colitis,” “nanocomposite,” “tumor microenvironment,” “exosome,” and “gut microbiota.” The intensity of these keywords remains variable, indicating that these research areas have garnered significant attention in recent years.

### 3.8 Research trends evolution

Author keywords are typically closely related to the content of publications and adequately reflect the primary research themes in a specific field (26). To conduct an in-depth analysis of the keywords provided by authors in the dataset and to identify popular research themes, we performed a visualization analysis using the bibliometrix package in R studio.

The thematic analysis reveals the clustering of author keywords and their interconnections. Each theme’s characteristics are determined by density and centrality attributes, with density represented on the vertical axis and centrality on the horizontal axis. Centrality reflects the degree of association between different themes, while density measures the cohesion among nodes (27). These two attributes evaluate the development potential and significance of the themes. **Supplementary Figure S6A** illustrates the thematic map of the application of nanomaterials in the field of CRC, divided into four quadrants (Q1 to Q4). According to the diagram, themes such as “exosome,” “biomarkers,” “diagnostic,” “anticancer,” “silver nanoparticles,” and “gold nanoparticles” are located in the Q1 quadrant, indicating that these research areas are not only well-developed in this field but also crucial for addressing critical challenges in CRC diagnosis and treatment. The Q2 quadrant includes themes like “hydrogel” and “targeted drug delivery,” which have reached a mature stage and show substantial potential for overcoming barriers to effective treatment, such as poor drug bioavailability and systemic toxicity, while also contributing to the sustainable development of nanomaterials in CRC.

Finally, we utilized R Studio to visualize and analyze the trends of the main keywords related to nanomaterials in CRC over the past decade (**Supplementary Figure S6B**). A line represents each topic, while circles indicate the most frequent year of appearance for each topic, with their sizes proportional to the frequency of the topics. The results suggest that “nanoparticles,” “colorectal cancer,” and “drug delivery” have been leading research trends in the past five years. The frequent occurrence of these themes demonstrates that they have received considerable attention and in-depth discussion. Notably, the emerging themes of “metal-organic frameworks” and “green” in 2024 have opened up several important directions in CRC research. The multifunctionality of MOFs supports the integration of diverse diagnostic and therapeutic strategies, providing new avenues for precision treatment and personalized medicine. Green synthesis methods not only foster the development of environmentally friendly and low-toxicity nanomaterials but also accelerate their clinical translation. Overall, these findings align with our previous analysis, further reinforcing the significance and development prospects of nanomaterials in CRC research.

## 4 Discussion

### 4.1 General information

This study conducted a systematic bibliometric analysis of the applications of nanomaterials in CRC from 2003 to 2024. Combining quantitative and qualitative methods, it analyzed 3,683 publications from 100 countries and regions, encompassing 3,721 institutions and 17,261 authors. This analysis thoroughly explored the current application status of nanomaterials in CRC treatment, filling a knowledge gap in this field and providing profound insights and guidance for future research.

The results indicate a significant increase in global publications in recent years, especially since 2018, suggesting a promising research outlook for nanomaterials in CRC. China ranks first globally both in publication volume and total citation counts, with six of the top ten institutions and seven of the ten most cited authors being Chinese scholars. This reflects China’s leading position in this research area, a success attributed to the close collaboration between Chinese universities and research institutions. Despite China’s outstanding performance in publication volume, the United States ranks first in citation rates per paper and academic centrality, highlighting its substantial influence in the international scholarly community. Therefore, it is recommended that the Chinese government and universities take measures to enhance the quality and impact of academic publications. Additionally, research institutions and scholars in various countries tend to collaborate with domestic peers; thus, it is suggested that international cooperation be strengthened to promote broader academic exchanges. Notably, Ramezani M and Abnous K from Mashhad University of Medical Sciences have excelled in publication volume and H-index, with research focusing on chemistry, pharmacology, and nanotechnology. Professor Siegel RL from the American Cancer Society is one of the most cited authors, indicating the significant impact of his research in academia. Furthermore, the number of journal publications and their co-citation frequency reflect their influence in the research field. The top ten journals and co-cited journals are primarily concentrated in the interdisciplinary area of nanoscience and medicine, dedicated to cross-disciplinary research between the two fields. High-quality journals such as ACS Nano, Biomaterials, Journal of Controlled Release, International Journal of Nanomedicine, and International Journal of Pharmaceutics have played an essential role in advancing the application of nanomaterials in CRC research.

### 4.2 Citation bursts

Citation bursts refer to a phenomenon where the citation frequency of literature suddenly increases within a short period, typically reflecting the research hotspots of a given time and the shift in research focus over different periods. The article “Cancer Nanotechnology: The Impact of Passive and Active Targeting in the Era of Modern Cancer Biology” (23) represents the most significant citation burst related to the topic. Mauro Ferrari et al. systematically review the lessons learned from the first commercialized nanomedicines, DOXIL and Abraxane, discussing the roles of passive and active targeting in cancer nanotherapy. Passive targeting utilizes the physical properties of nanocarriers, such as size and surface characteristics, to achieve accumulation in solid tumors. In contrast, active targeting involves modifying the surface of nanocarriers with specific ligands to target specific cancer cells. These discussions deepen our understanding of the design and development of therapeutic nanomaterials and indicate directions for developing multifunctional nanocarriers that enhance efficacy while reducing toxicity.

It is particularly noteworthy that five papers are currently experiencing a citation explosion. These works not only highlight significant advancements in the field but also reflect the current hot topics in research. Analysis reveals that the themes they focus on are primarily concentrated in several key areas: (1) The application of different types of nanomaterials in drug delivery for CRC. For instance, the study by Elaheh Entezar-Almahdi et al. provides a detailed exploration of PNPs, liposomes, and hydrogels as nanocarriers for delivering 5-FU. This research highlights the unique advantages of these nanocarriers in enhancing the efficacy of 5-FU while reducing its toxicity (28, 29). (2) The application of different types of nanomaterials in diagnosing and imaging CRC. Metal nanoparticles (MNPs) have been widely studied for precise tumor imaging, improving imaging clarity and sensitivity, which helps doctors detect tumors earlier and assess their progression. Additionally, PNPs and liposomes play a significant role in drug delivery and provide visual feedback when combined with imaging techniques, making treatment plans more scientific and reasonable. The application of photosensitizer nanoparticles in PDT has also gained attention, offering new strategies for treating CRC (11, 30). (3) The significant advantages of nanomaterials. Firstly, nano-drug delivery systems enable precise and direct action of drugs on tumor cells, thereby minimizing damage to healthy cells. Secondly, nanomaterials can enhance the solubility and bioavailability of drugs, which improves therapeutic efficacy and ultimately benefits patient prognosis. Furthermore, nanomaterials can sustain drug release through controlled release mechanisms, achieving long-term therapeutic effects without frequent administration. Additionally, nanomaterials can effectively reduce the toxic side effects of chemotherapeutic agents, significantly improving patient tolerance during treatment and alleviating chemotherapy-related discomfort (30) (31). (4) The role of nanomaterials in overcoming tumor resistance (31). They primarily overcome resistance through multiple mechanisms: firstly, nanomaterials target specific surface receptors on tumor cells, bypassing the tumor cells’ resistance mechanisms and thereby enhancing drug endocytosis and accumulation. Secondly, nanomaterials improve the concentration of drugs in the tumor microenvironment while reducing their distribution in normal tissues by altering pharmacokinetic properties such as drug solubility, stability, and circulation time, thus overcoming resistance. Moreover, nanomaterials can be designed to deliver siRNA or other gene inhibitors to reduce the expression of genes associated with resistance, thereby increasing tumor cell sensitivity to chemotherapeutic agents. Finally, nanomaterials can co-deliver chemotherapeutic agents and resistance-reversing agents, further inhibiting resistance through synergistic effects. (5) Design and development of nanomaterials and challenges in clinical translation (29). Researchers optimize the performance of nanomaterials through meticulous engineering, focusing on parameters such as size, shape, and surface properties to enhance their effectiveness in drug delivery applications. However, even though nanomaterials demonstrate favorable performance in laboratory experiments, they still face numerous challenges during the clinical translation process, including pharmacokinetics, toxicity, standardization of production, and scalability. To overcome these obstacles, it is essential to refine nanomaterial formulations’ design and manufacturing processes and conduct additional preclinical studies and clinical trials to validate their safety and efficacy. Moreover, close collaboration with regulatory authorities is crucial to ensure the smooth progression of clinical translation.

### 4.3 Research hotspots

In the current study, we conducted a comprehensive analysis of the applications of nanomaterials in CRC through keyword clustering, ultimately resulting in four clusters that identify the research hotspots and development trends in this field.

**Cluster 1:** The application of nanomaterials in diagnosing and treating CRC and their impact on the biological behavior of CRC cells. In this cluster, keywords diagnosis, biomarkers, assay, antibody, protein, receptor, and CD44 can be interconnected to indicate the application of nanomaterials in the diagnosis and treatment of CRC. Keywords metastasis, growth, migration, and invasion can also be interrelated to represent the influence of nanomaterials on the biological behavior of CRC cells. Additionally, Keywords stem cells, exosome, and dendritic cells can be associated as well, they play significant roles in tumor progression and can interact with nanomaterials, providing new directions for the diagnosis and treatment of CRC.

Research related to the application of nanomaterials in CRC primarily focuses on nano-drug delivery systems. These studies mainly achieve anti-tumor effects by influencing CRC cells’ proliferation, growth, metastasis, and invasion capabilities or by regulating specific signaling pathways.

The resistance of CRC stem cells to conventional chemotherapy drugs, such as 5-FU and oxaliplatin, is one of the significant reasons for treatment failure and recurrence (32). Moreover, CRC stem cells exhibit a high capacity for self-renewal and unlimited proliferation potential (33). Increasing evidence suggests that CRC stem cells are key drivers of tumor initiation, progression, metastasis, and recurrence (34). Targeting the biomarkers on the surface of CRC stem cells, such as CD44 and CD133, using nanomaterials is a potentially effective strategy for treating CRC. Xu et al. found that AuNPs can serve as effective carriers to specifically target CD44-positive CRC stem cells, delivering curcumin to activate FOXO1, thereby inhibiting CRC proliferation and malignant progression (35). Additionally, anti-CD133 antibody-modified SN-38 nanoparticles can selectively act on CD133-positive CRC stem cells, inhibiting their proliferation and inducing apoptosis, thus providing new possibilities for targeted therapy of CRC (36).

Exosomes, membrane-bound vesicles secreted by cells, play a crucial role in CRC occurrence, development, diagnosis, and treatment. Their potential as therapeutic targets and biomarkers is increasingly gaining attention. Exosomes exhibit excellent targeting capabilities, capable of transporting bioactive substances such as nucleic acids and proteins and effectively delivering drugs or non-coding RNAs to CRC cells (37, 38), further impacting the biological behavior of tumor cells. Combining exosomes with nanomaterials, followed by surface modification to achieve active targeting, can further enhance their targeting capability and therapeutic efficacy. For instance, Zhang et al. loaded mRNA of the m6A demethylase ALKBH5 into exosome-lipid hybrid nanoparticles and found that ALKBH5 overexpression can suppress the proliferation and metastasis of CRC cells by regulating m6A modifications (39). Furthermore, exosomes serve as liquid biopsy biomarkers for CRC, allowing researchers to achieve early diagnosis by detecting specific exosomes secreted by tumor cells. Nanotechnology offers new opportunities for the selective capture and separation of exosomes. By combining exosomes with nanomaterials, novel diagnostic platforms can be developed to significantly enhance the sensitivity and specificity of detection (40, 41). Specifically, nano-sensors based on exosomes and AuNPs have been applied to the early detection of CRC (42). Researchers modified AuNPs to enable their specific recognition and effective capture of exosomes released by CRC cells. This interaction generates corresponding color or fluorescent changes, allowing for the quantitative analysis of exosome content in samples and achieving highly sensitive detection.

**Cluster 2:** Nano-drug delivery system and their characteristics and common nanocarriers for CRC. In this cluster, keywords nanoparticle, drug delivery, oral delivery, release, pH sensitivity, bioavailability, stability, antitumor activity, and efficacy can be associated to indicate nano-drug delivery systems based on nanomaterials and their characteristics. Meanwhile, keywords liposomes, polymeric nanoparticles, microspheres, curcumin, camptothecin, and cisplatin represent the primary nanocarriers used for CRC and the common drugs they carry.

Nanomaterials typically possess small sizes ranging from 1 nm to 100 nm and high specific surface areas, with their size, shape, and surface characteristics precisely tunable to enhance drug loading capacity and release efficiency. Nano-drug delivery systems aim to improve drug retention, accumulation, penetration, and targeted cellular uptake at tumor sites by loading drugs onto these nanomaterials, thus achieving more precise drug release and delivery.

In recent years, targeted oral nano-drug delivery systems have garnered widespread attention. Researchers have designed specifically targeted nanomaterials through surface modification techniques to effectively deliver drugs to tumor cell membrane receptors, intestinal epithelial cells, and intestinal mucosa (43). By conjugating specific ligands or absorption enhancers, the affinity of nanomaterials for target cells can be significantly increased, thereby enhancing drug accumulation and absorption at the target sites. This approach not only improves the bioavailability and stability of oral medications but also effectively mitigates systemic side effects. The main pathways of targeted oral nano-drug delivery systems in CRC treatment include pH-based delivery, enzyme-based delivery, time-controlled delivery, and gastrointestinal pressure-based delivery. Furthermore, various methods, including prodrug systems, hydrogel systems, and microbiota-triggered systems, have also been explored (44). Recent studies frequently integrate various techniques to address the limitations of individual methods, thereby achieving effective drug delivery. For instance, Abd Elhamid, A. S. et al. have pioneered a dual-responsive lactoferrin nanostructured microsphere that responds to both temperature and pH, establishing a promising oral targeted delivery system for CRC that significantly improved targeting and therapeutic effects in the intestinal environment (45). Ma et al. developed a pH and enzyme dual-responsive hydrogel loaded with 5-FU based on ozalazine derivatives, which enables specific drug release in response to pH changes and intestinal enzyme activity within the intestinal environment (46).

According to our analysis, liposomes, PNPs, and polymeric microspheres (PMs) are commonly used nano-drug carriers for CRC diagnosis and treatment.

Liposomes are one of the most widely used nanocarriers. They not only achieve drug accumulation through passive targeting mechanisms (e.g., utilizing characteristics of tumor vasculature) but can also enhance therapeutic effects via active targeting mechanisms (e.g., binding to specific antigens, antibodies, or enzymes). Liposomes exhibit good biocompatibility and can enable sustained drug release, but they have poor stability and are easily affected by serum protein adsorption. Researchers have developed novel liposomal materials recently, demonstrating significant advantages through targeted modifications and combination therapy strategies. For example, London M loaded panitumumab and cetuximab into liposomes to achieve more precise targeting of EGFR (47). Additionally, liposomes can serve as carriers for other therapeutic modalities, combined with immunotherapy, targeted therapy, PDT, and RNA interference therapy.

PNPs are composed of biodegradable polymers, which possess excellent biocompatibility and degradability, allowing for prolonged retention time in the body through sustained release, achieving enhanced efficacy and reduced toxicity (11). The high permeability and retention of CRC tissues facilitate the targeted accumulation of nanomaterials at tumor sites, further increasing drug concentration (6). Udompornmongkol et al. effectively encapsulated the anticancer drug curcumin in PNPs utilizing the EPR effect. These nanomaterials exhibited up to 95% encapsulation efficiency and resistance to gastric fluid or intestinal enzyme hydrolysis, demonstrating more potent antitumor activity against CRC *in vitro* than free curcumin (48).

PMs and PNPs represent the same type of material at different scales. Compared to other nanomaterials, PMs exhibit higher drug-loading capacity, excellent biocompatibility, strong permeability, and outstanding multifunctionality. The internal cavities of PMs can accommodate large amounts of anticancer drugs, with drug loading capacities reaching up to 15-20%, significantly higher than that of other nanocarriers (49, 50). The size of microspheres can be precisely controlled by adjusting the preparation process parameters (e.g. emulsification speed and polymer concentration), aiding in improving drug accumulation in CRC tissues (51). Furthermore, PMs can simultaneously load multiple functional components, such as anticancer drugs, imaging agents, and targeting ligands, thereby achieving integrated diagnosis and therapy.

**Cluster 3:** Applications of different types of nanomaterials and their cytotoxicity issues, as well as the application of green synthesis methods in preparation. In this cluster, keywords gold nanoparticles, silver nanoparticles, nanocomposite, graphene oxide, and magnetic nanoparticles are interrelated, indicating the applications of different types of nanoparticles in diagnosing and treating CRC. Keywords cytotoxicity and oxidative stress are connected, representing the cytotoxicity concerns associated with the nanomaterials used in CRC. Meanwhile, keywords green synthesis, antioxidant, extract, and fluorescence are linked, suggesting the application of green synthesis methods in the preparation of nanomaterials.

AuNPs and AgNPs are the most extensively studied and well-established metal nanomaterials in the field of CRC. Their unique optical and surface chemical properties enable their use in optical imaging and targeted imaging for CRC diagnosis (52, 53). They facilitate targeted drug delivery through surface modification with targeting ligands in therapeutic applications (54). AuNPs exhibit higher photothermal conversion efficiency under near-infrared light irradiation, which can effectively kill tumor cells and enable photothermal therapy (PTT) (55), whereas AgNPs possess antibacterial properties, effectively preventing and controlling infections at the surgical sites of CRC (56).

Traditional chemical synthesis methods often require toxic, strong reducing agents and organic solvents, which lead to environmental pollution and pose risks to biological safety. As a result, green synthesis methods have garnered increasing attention. This approach employs plant extracts (such as tea polyphenols, citric acid (57)), microorganisms (such as bacteria, yeast, and algae (58–60)), or enzymes (such as glucose oxidase, lactate dehydrogenase (61, 62)) as reducing agents and stabilizers, thereby avoiding harmful chemicals and providing more environmentally friendly and sustainable alternatives. Furthermore, this method imparts good biocompatibility and bioactivity to AuNPs and AgNPs (63, 64). For example, N. González-Ballesteros et al. successfully synthesized AuNPs with excellent antitumor activity using the brown algae Cystoseira baccata through a green synthesis method. These nanomaterials exhibited a concentration-dependent inhibitory effect on CRC cell lines HT-29 and SW480, showing that cell viability significantly decreased as the concentration of AuNPs increased (65).

Nanocomposites are novel materials composed of two or more different components, with at least one component having a nanoscale dimension (1-100 nanometers). These nanoscale components impart unique physical, chemical, and biological properties to the composite material. Typically, nanocomposites consist of matrix material (such as polymers, metals, or carbon materials), nanofillers (such as nanoparticles or nanotubes), and functional components (such as targeting ligands, drugs, or diagnostic probes). In recent years, nanocomposites synthesized from MNPs and other materials, such as NPs or graphene oxides, have become a hotspot in CRC research. These composites effectively integrate multiple functions, such as diagnostic imaging, targeted drug delivery, and PTT, while optimizing the inherent characteristics of each component for more precise and efficient cancer treatment. Particularly, magnetic gold nanoparticles (MGNPs) exhibit unique physicochemical properties that enhance various cancer diagnostic techniques, including MRI, X-ray computed tomography, Raman imaging, and photoacoustic imaging (66), and are also effective for drug delivery and therapeutic modalities like plasmonic photothermal and photodynamic therapies (67). Moreover, composites of MNPs with graphene oxide can be utilized for detecting the biomarkers of CRC, such as CEA and CA19-9, facilitating early diagnosis. Such multifunctional nanocomposites provide an integrated approach to diagnostics and therapeutics, commonly referred to as “theranostics.”

Although nanocomposites have been extensively researched for CRC therapy, cytotoxicity remains a critical determinant of efficacy and safety. Various factors influence cytotoxicity, including the type of nanomaterial composition, particle size, surface properties, drug release rate, and target cell types. *In vitro* and *in vivo* toxicity studies indicate that pure AuNPs and NPs exhibit relatively low toxicity (68, 69). However, our understanding of the toxic effects of MGNP hybrids in biological systems is still limited. Typically, nanoparticles smaller than 50 nanometers with a positive charge are more readily taken up by cells, which may result in increased cytotoxicity. Furthermore, the drug release rate of nanomaterials is crucial for their anticancer efficacy; rapid release can lead to intense toxicity within a short time, while sustained release can facilitate prolonged therapeutic effects. Recent research suggests that optimizing the structural composition of nanocomposites, controlling smart drug release, and conducting functional surface modifications, combined with synergistic therapies, can effectively reduce cytotoxicity. For instance, Bardania et al. developed a folate-modified graphene nanocomposite that loaded Cur or 5-FU onto the nanomaterials, enhancing the targeting capability of the drug in human CRC cells (HT-29) and significantly improving its anticancer effects. Experimental studies demonstrate that folate-modified nanomaterials show better cytotoxic profiles than their unmodified counterparts (70).

**Cluster 4:** Resistance mechanisms in CRC treatment and various strategies utilizing nanomaterials to overcome resistance. In this cluster, keywords resistance, chemotherapy, radiotherapy, and immunotherapy are interconnected, reflecting the issue of drug resistance encountered in various CRC therapies. Keywords death mechanisms, apoptosis, autophagy, and ferroptosis are also interrelated, illustrating how nanomaterials can target and regulate tumor cell death mechanisms to overcome resistance. Furthermore, keywords microenvironment and gut microbiota are closely linked, demonstrating the potential of nanomaterials to improve the tumor microenvironment and modulate the gut microbiota to combat resistance.

Mechanisms of resistance often constrain the efficacy of conventional drugs. However, nanomaterials can effectively overcome resistance through multiple pathways, including targeting tumor cell death, improving tumor microenvironment, and modulating gut microbiota. Therefore, utilizing nanomaterials to address the issue of CRC resistance has become a major research focus, with the potential to enhance CRC treatment outcomes significantly.

Firstly, nanomaterials can target and modulate the death mechanisms of tumor cells, including apoptosis, autophagy (71), and ferroptosis (72), thereby effectively overcoming resistance. Among these pathways, autophagy-dependent ferroptosis is regarded as a novel target for cancer treatment. Kim et al. developed a novel ultra-small-scale nanoparticle that accumulates in the tumor microenvironment under nutrient-deprived conditions, releasing iron ions through autophagy, resulting in oxidative stress and lipid peroxidation, ultimately inducing ferroptosis to kill tumor cells. This nanomaterial has demonstrated significant anti-tumor activity across various drug-resistant tumor models (73).

Secondly, drugs designed using nanocarrier systems to target and modulate the tumor microenvironment, such as those aimed at regulating tumor vasculature and reducing hypoxia, can significantly enhance drug efficacy and overcome resistance (25, 74). Tumor angiogenesis is considered a critical factor in tumor progression and the development of resistance, and the “leaky” characteristics of tumor blood vessels facilitate the accumulation of nanocarriers (75, 76). Studies have shown that albumin nanoparticles loaded with bevacizumab can selectively accumulate in tumor blood vessels, effectively inhibit tumor angiogenesis, improve tumor vascular function, and enhance the oxygenation status of tumor tissues. In CRC animal models, this nanocarrier exhibited superior anti-tumor efficacy compared to free bevacizumab, significantly overcoming CRC resistance (77). Additionally, hypoxic conditions in the tumor microenvironment tend to lead to tumor cell resistance to chemoradiotherapy. Nanomaterials can carry oxygen or redox-sensitive drugs to improve the hypoxic microenvironment of tumors, thereby enhancing drug efficacy and overcoming resistance (78). For example, HIF-1α is a critical regulatory factor contributing to drug resistance in tumor cells under hypoxic conditions. Chen et al. developed a novel nanocarrier that encapsulates components capable of inhibiting the HIF-1α signaling pathway, aiming to block this pathway to enhance the cytotoxic effects of chemotherapeutic agents (79).

Recent studies have shown that the imbalance of gut microbiota is one of the important factors contributing to drug resistance in CRC (80). Therefore, utilizing nanotechnology to modulate gut microbiota to reverse tumor resistance has become a highly researched topic. Researchers have developed various targeted nanocarriers for the gut to selectively regulate the growth of harmful and beneficial bacteria (81). Wang et al. designed a type of biologically membrane-coated nanoparticles (BMNPs) composed of lipids, polysaccharides, and proteins. By mimicking the structure of bacterial biofilms and utilizing their adhesive and permeable properties, BMNPs efficiently traverse the intestinal mucosal barrier, significantly prolonging their retention time in the gut. Furthermore, BMNPs can selectively inhibit the growth of harmful bacteria while promoting the proliferation of beneficial bacteria. After treatment, BMNPs significantly increased the diversity of the gut microbiota, achieving a growth rate of 1.6 times compared to the control group, successfully reversing the tumor-associated gut dysbiosis and overcoming drug resistance (82).

Future research should further optimize the targeting and biocompatibility of nanomaterials and explore their synergistic effects with other therapeutic modalities, aiming to provide breakthroughs in overcoming resistance.

### 4.4 Emerging frontiers

**Figure 8:**
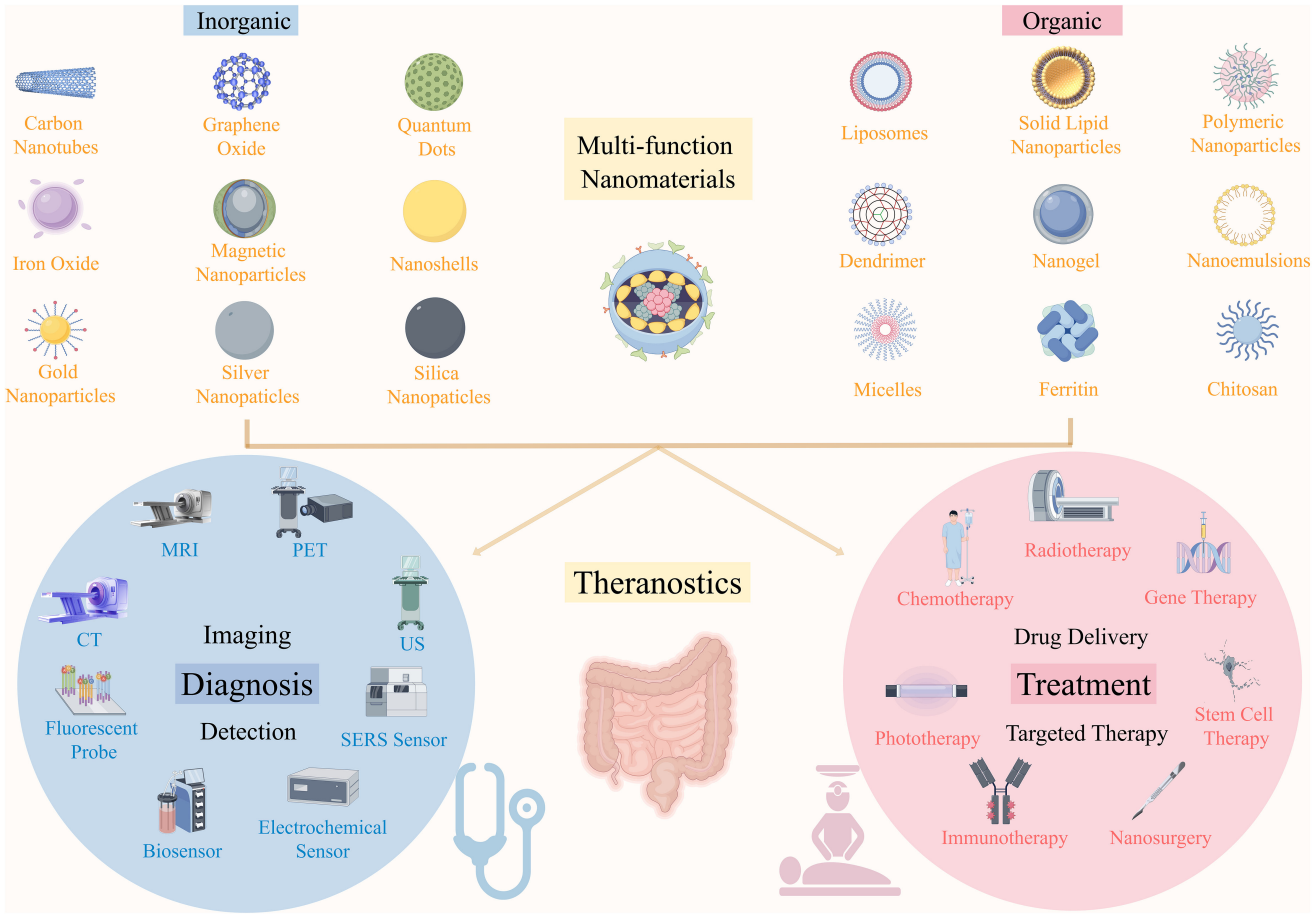
The application of different types of nanomaterials in the diagnosis and treatment of CRC.

Nanomaterials can be classified into organic and inorganic categories, both of which play significant roles in CRC diagnosis and treatment (**Figure 8**). Inorganic nanomaterials, including carbon-based nanomaterials (carbon nanotubes, graphene oxide), metal nanoparticles (gold nanoparticles, silver nanoparticles), quantum dots, and magnetic nanoparticles, are particularly suitable for long-term tracking and imaging *in vivo* due to their excellent stability, providing important support for precise diagnosis of CRC. These materials are pivotal in imaging techniques, such as CT, MRI, PET, and ultrasound, as well as in early detection methods like fluorescence probes and sensors. In contrast, organic nanomaterials, such as lipid-based nanomaterials (liposomes, solid lipid nanoparticles), polymer-based nanomaterials (polymeric nanoparticles, dendrimers, nanogels), surfactant-based nanomaterials (nanoemulsions, micelles), and biomolecule-based nanomaterials (ferritin, chitosan), demonstrate enormous potential in drug delivery and targeted therapy due to their good biocompatibility. They have shown promising applications in chemotherapy, radiotherapy, immunotherapy, and gene therapy (83). Although nanomaterials can be categorized into organic and inorganic types based on their composition, their applications are not limited to a single function. Many organic nanomaterials play significant roles not only in CRC treatment but also in diagnosis. For instance, liposomes, PNPs, and micelles can effectively enhance imaging effects and improve early tumor detection when combined with imaging probes (10).Moreover, surface modification or targeted functional design significantly enhances diagnostic specificity and sensitivity. Meanwhile, inorganic nanomaterials, such as AuNPs, carbon nanotubes, and graphene oxide, have shown considerable therapeutic potential in fields such as PTT, magnetic hyperthermia, and targeted drug delivery, which help improve CRC treatment outcomes while minimizing side effects (84).

In recent years, researchers have been actively exploring the combination of various types of nanomaterials with biomolecules and anticancer drugs to develop multifunctional nanocomposites that integrate diagnostic and therapeutic capabilities, aiming to construct a “theranostics” platform (85).These materials can leverage the stability and imaging capabilities of inorganic nanomaterials, as well as the biocompatibility and targeting characteristics of organic nanomaterials, by carrying anticancer drugs to achieve precise diagnosis, targeted treatment, and combined therapy for CRC, thereby advancing the development of personalized medicine.

On the one hand, multifunctional nanocomposites play a crucial role in early detection and imaging diagnosis of CRC. They overcome the limitations of traditional screening methods, such as colonoscopy and fecal occult blood tests, offering superior sensitivity for early detection (86).These materials utilize their exceptional surface effects to amplify signals, enabling the rapid detection of trace cancer-related molecules, thereby facilitating more precise early screening (87).Furthermore, the convergence of diverse imaging techniques including optical imaging, MRI, and fluorescence imaging substantially boosts the capability of clinicians to conduct a thorough evaluation of tumor properties. On the other hand, multifunctional nanomaterials offer significant advantages in drug delivery and targeted therapy. Their small particle size and large surface area enable efficient drug loading and crossing of biological barriers, ensuring precise delivery of therapeutics to tumor sites, significantly improving therapeutic efficacy (88).By modifying the surface of the carriers with specific ligands, targeted delivery can be achieved, allowing selective identification of receptors on tumor cells, thus reducing damage to healthy tissues and minimizing side effects. Moreover, the structure and surface characteristics of these materials can be designed to respond to changes in the tumor microenvironment, enabling controlled drug release and further enhancing treatment outcomes. More importantly, multifunctional nanocomposites can simultaneously carry multiple therapeutic agents, facilitating combination therapy, and can be integrated with imaging probes for real-time monitoring, providing further options for personalized treatment (89).So far, the FDA has approved three nanomedicines for the treatment of CRC: Onivyde (irinotecan liposome injection), Injectafter Ferinject (Iron carboxymaltose colloid), and Doxil synergized with mAbs (Liposomal doxorubicin, anti-PD-1 and CTLA-4 mAbs). Furthermore, clinical trials for multifunctional nanomaterials in the treatment of CRC are also being widely conducted (**Supplementary Table S4**) (30, 90–94).

Nanocomposites made from MOFs have shown great promise in theranostics research. For diagnostics, MOFs offer remarkable benefits due to their extremely high specific surface area and adjustable pore size, which allow them to accommodate multiple drugs. Additionally, they can be effectively integrated with imaging probes for image-guided therapies, addressing one of the major challenges in CRC diagnosis—the difficulty in achieving early and accurate detection. By enhancing imaging contrast while simultaneously delivering diagnostic agents, MOFs improve the specificity and sensitivity of CRC detection. For therapeutics, MOFs also support PDT and PTT, enabling the generation of reactive oxygen species and heat under specific wavelengths of light through the delivery of photosensitizing drugs and photothermal conversion materials, thereby directly eliminating cancer cells (95). Furthermore, MOFs can simultaneously deliver chemotherapy drugs or be combined with radiotherapy and immunotherapy, providing a multifaceted and integrated treatment strategy (96, 97). Their multifunctionality is particularly promising for overcoming the challenges of heterogeneity and drug resistance in CRC, which hinder long-term therapeutic success. Finally, the unique properties of MOFs also facilitate real-time monitoring of treatment effectiveness, enabling clinicians to track the dynamic changes of MOFs within the body, thus providing critical data for informed decision-making. This capability is vital for personalized medicine, as it allows for treatments to be tailored to the evolving behavior of the tumor and the patient’s individual therapeutic needs.

As an emerging research field, green synthetic methods have garnered widespread attention due to their sustainable preparation approaches. These methods offer significant advantages, ensuring both the biocompatibility and environmental safety of nanomaterials, facilitating their metabolism and excretion within the body, and effectively addressing potential toxicity and long-term safety concerns in clinical applications. Green-synthesized nanomaterials, compared to those produced by traditional chemical synthesis, exhibit broad application prospects in the early diagnosis, targeted therapy, and prognostic monitoring of CRC. By optimizing the properties and biological behavior of these nanomaterials and conducting in-depth preclinical and clinical studies, they are expected to provide innovative solutions for precision diagnosis and treatment of CRC, thus facilitating the translation of nanomedicine technologies from the laboratory to clinical practice.

Overall, despite the tremendous potential of nanomaterials in CRC treatment, several challenges remain, including toxicity, *in vivo* behavior, quality control in preparation, and the complexities of clinical translation, all of which hinder their widespread clinical application. Continuous multidisciplinary cooperation is essential to overcome these bottlenecks, integrating expertise from materials science, clinical medicine, and the industrial sector. This collaborative effort is crucial for the ongoing innovation and development of safer and more effective nanomaterials. In this context, multifunctional nanomaterials and their green synthesis methods offer practical solutions, paving the way for more effective and personalized approaches to CRC diagnosis and treatment.

## 5 Innovation and limitation

Compared to traditional methods, visualization tools like CiteSpace, VOSviewer, and RStudio provide a more comprehensive understanding of the research dynamics of nanomaterials in CRC. CiteSpace excels in co-citation analysis and knowledge mapping, effectively identifying key terms and outlining the development trajectory of research areas. VOSviewer, with its advanced network visualization capabilities, offers a clear representation of relationships between research topics, helping researchers intuitively grasp their connections and structure. RStudio, in turn, provides robust support for data analysis and customized visualizations, allowing for the flexible creation of various charts tailored to specific research needs. Together, these three tools enable a deep analysis of bibliometric data and a clear visualization of research trends, emerging hotspots, and future directions in the field.

However, our study may suffer from incomplete literature coverage. First, our analysis relied exclusively on the WOSCC, which, while a comprehensive and authoritative source, represents only a subset of global literature. As a result, relevant publications from other major databases, such as PubMed or EMBASE, were excluded. These databases offer extensive clinical and biomedical literature, providing insights beyond our current analysis’s scope. Their exclusion may have led to a partial view of the research landscape, especially in fields where clinical or biomedical studies are prevalent. Additionally, our search was limited to English-language publications, which may have inadvertently excluded valuable research published in other languages. Furthermore, recently published high-quality papers may not have received sufficient attention due to low citation rates, underscoring the importance of regularly updating research.

To address these limitations, future studies could broaden their scope by incorporating additional databases such as PubMed, EMBASE, Scopus, and others that index a wider range of disciplines and languages. This would offer a more comprehensive overview of the literature, particularly for interdisciplinary topics or those focused on clinical and biomedical research. Moreover, future research could incorporate clinical application data or integrate real-world evidence to complement bibliometric analyses, thereby enhancing the comprehensiveness and representativeness of the study.

## 6 Conclusions

In this study, we performed a comprehensive bibliometric analysis to assess the research trends and advancements in the application of nanomaterials for CRC. Our results reveal the rapid growth of this field, with China and the United States at the forefront of global research efforts. To further accelerate progress, fostering international collaboration among research institutions and scientists is crucial. Additionally, we identify key research hotspots, emerging trends, and the field’s major challenges. With the interdisciplinary integration of medicine, materials science, and chemistry, the multifunctionality and precision design of nanomaterials have demonstrated significant potential in personalized and precision medicine. Cancer biomarkers provide pivotal targets for designing nanomaterials that specifically address molecular features in CRC cells. Through surface modifications, the targeting efficiency can be enhanced, improving bio-distribution and tumor penetration *in vivo*. Multifunctional nanomaterials, integrating both diagnostic and therapeutic functions, emerge as a highly promising category of “theranostic” agents. These nanomaterials not only enable efficient drug delivery but can also be coupled with imaging technologies to allow for real-time monitoring of treatment progress. Moreover, they can be engineered to respond dynamically to changes in the tumor microenvironment, releasing agents at the precise target site. This approach maximizes therapeutic efficacy while minimizing toxicity and other adverse effects, ultimately improving patient outcomes. However, the clinical application of nanomaterials still faces a series of technical and regulatory challenges, including safety, toxicity, and production standardization. Future research should focus on optimizing the targeting precision and multifunctionality of nanomaterials while simultaneously ensuring their safety and biocompatibility. This will provide more efficient, precise, and sustainable therapeutic solutions, driving the ongoing advancement of nanomaterials in personalized and precision medicine.

## Data Availability

The datasets presented in this study can be found in online repositories. The names of the repository/repositories and accession number(s) can be found in the article/**Supplementary Material**.

## Supplementary material

Supplementary Figure 1 Research on nano-drug delivery systems and medical imaging in different types of cancer.

Supplementary Figure 2 Networks of country cooperation.

Supplementary Figure 3 Visual mapping of the authors and co-cited authors. **(A)** Networks of author cooperation; **(B)** Networks of co-cited authors.

Supplementary Figure 4 Visual mapping of the journals and co-cited journals. **(A)** Density map of journal publications; **(B)** Networks of co-cited journals.

Supplementary Figure 5 Visual mapping of the co-cited references. **(A)** Networks of co-cited references; **(B)** Clustering of co-cited references; **(C)** Peak map of clustering of co-cited references over time.

Supplementary Figure 6 Research trends in the field. **(A)** Keyword-based research trend quadrant chart (2003-2024); **(B)** Trend topics of the research field (2014 to 2024).

## References

[1] Arnold M Abnet CC Neale RE Vignat J Giovannucci EL McGlynn KA . Global burden of 5 major types of gastrointestinal cancer. Gastroenterology. (2020) 159:335–349.e315. doi: 10.1053/j.gastro.2020.02.068 32247694 PMC8630546

[2] Siegel RL Wagle NS Cercek A Smith RA Jemal A . Colorectal cancer statistics, 2023. CA Cancer J Clin. (2023) 73:233–54. doi: 10.3322/caac.21772 36856579

[3] Keum N Giovannucci E . Global burden of colorectal cancer: emerging trends. risk factors and prevention strategies. Nat Rev Gastroenterol hepatology. (2019) 16:713–32. doi: 10.1038/s41575-019-0189-8 31455888

[4] Xi Y Xu P . Global colorectal cancer burden in 2020 and projections to 2040. Trans Oncol. (2021) 14:101174. doi: 10.1016/j.tranon.2021.101174 PMC8273208 34243011

[5] Arruebo M Vilaboa N Sáez-Gutierrez B Lambea J Tres A Valladares M . Assessment of the evolution of cancer treatment therapies. Cancers (Basel). (2011) 3:3279–330. doi: 10.3390/cancers3033279 PMC3759197 24212956

[6] Maeda H Nakamura H Fang J . The EPR effect for macromolecular drug delivery to solid tumors: Improvement of tumor uptake. lowering of systemic toxicity. and distinct tumor imaging in *vivo* . Advanced Drug delivery Rev. (2013) 65:71–9. doi: 10.1016/j.addr.2012.10.002 23088862

[7] Patel NR Pattni BS Abouzeid AH Torchilin VP . Nanopreparations to overcome multidrug resistance in cancer. Adv Drug Delivery Rev. (2013) 65:1748–62. doi: 10.1016/j.addr.2013.08.004 PMC3840079 23973912

[8] Housman G Byler S Heerboth S Lapinska K Longacre M Snyder N . Drug resistance in cancer: an overview. Cancers (Basel). (2014) 6:1769–92. doi: 10.3390/cancers6031769 PMC4190567 25198391

[9] Bousbaa H . Novel anticancer strategies. Pharmaceutics. (2021) 13:275. doi: 10.3390/pharmaceutics13020275 33670469 PMC7922003

[10] Brar B Ranjan K Palria A Kumar R Ghosh M Sihag S . Nanotechnology in colorectal cancer for precision diagnosis and therapy. Front Nanotechnology. (2021) 3:699266. doi: 10.3389/fnano.2021.699266

[11] Pavitra E Dariya B Srivani G Kang SM Alam A Sudhir PR . Engineered nanoparticles for imaging and drug delivery in colorectal cancer. Semin Cancer Biol. (2021) 69:293–306. doi: 10.1016/j.semcancer.2019.06.017 31260733

[12] Badran MM Mady MM Ghannam MM Shakeel F . Preparation and characterization of polymeric nanoparticles surface modified with chitosan for target treatment of colorectal cancer. Int J Biol Macromol. (2017) 95:643–9. doi: 10.1016/j.ijbiomac.2016.11.098 27908720

[13] Wang B Hu S Teng Y Chen J Wang H Xu Y . Current advance of nanotechnology in diagnosis and treatment for Malignant tumors. Signal Transduct Target Ther. (2024) 9:200. doi: 10.1038/s41392-024-01889-y 39128942 PMC11323968

[14] Gaviria-Marin M Merigó JM Baier-Fuentes H . Knowledge management: A global examination based on bibliometric analysis. Technological Forecasting Soc Change. (2019) 140:194–220. doi: 10.1016/j.techfore.2018.07.006

[15] Chen CM . Visualizing and exploring scientific literature with citeSpace: an introduction. In: Proceedings of the 2018 Conference on Human Information Interaction & Retrieval (CHIIR '18). Association for Computing Machinery, New York, NY, USA (2018). p. 369–70. doi: 10.1145/3176349.3176897

[16] Yan SJ Chen M Wen J Fu WN Song XY Chen HJ . Global research trends in cardiac arrest research: a visual analysis of the literature based on CiteSpace. World J Emergency Med. (2022) 13:290–6. doi: 10.5847/wjem.j.1920-8642.2022.071 PMC9233974 35837560

[17] van Eck N Waltman L . Software survey: VOSviewer, a computer program for bibliometric mapping. Scientometrics. (2010) 84:523–38. doi: 10.1007/s11192-009-0146-3 PMC2883932 20585380

[18] Van Eck NJ Waltman L . Citation-based clustering of publications using CitNetExplorer and VOSviewer. Scientometrics. (2017) 111:1053–70. doi: 10.1007/s11192-017-2300-7 PMC5400793 28490825

[19] Radha L Arumugam J . The research output of bibliometrics using bibliometrix R package and VOS viewer. Shanlax Int J Arts. Sciecne Humanities. (2021) 9:44–9. doi: 10.34293/sijash.v9i2.4197.

[20] Siegel RL Miller KD Fuchs HE Jemal A . Cancer statistics, 2021. CA Cancer J Clin. (2021) 71:7–33. doi: 10.3322/caac.21654 33433946

[21] Dekker E Tanis PJ Vleugels JLA Kasi PM Wallace MB . Colorectal cancer. Lancet (London England). (2019) 394:1467–80. doi: 10.1016/s0140-6736(19)32319-0 31631858

[22] Xie YH Chen YX Fang JY . Comprehensive review of targeted therapy for colorectal cancer. Signal transduction targeted Ther. (2020) 5:22. doi: 10.1038/s41392-020-0116-z PMC7082344 32296018

[23] Bertrand N Wu J Xu X Kamaly N Farokhzad OC . Cancer nanotechnology: the impact of passive and active targeting in the era of modern cancer biology. Advanced Drug delivery Rev. (2014) 66:2–25. doi: 10.1016/j.addr.2013.11.009 PMC4219254 24270007

[24] Wilhelm S Tavares AJ Dai Q Ohta S Audet J Dvorak HF . Analysis of nanoparticle delivery to tumours. Nat Rev Materials. (2016) 1:16014. doi: 10.1038/natrevmats.2016.14

[25] Cisterna BA Kamaly N Choi WI Tavakkoli A Farokhzad OC Vilos C . Targeted nanoparticles for colorectal cancer. Nanomedicine (Lond). (2016) 11:2443–56. doi: 10.2217/nnm-2016-0194 PMC5619175 27529192

[26] Song Y Chen XL Hao TY Liu ZN Lan ZX . Exploring two decades of research on classroom dialogue by using bibliometric analysis. Comput Education. (2019) 137:12–31. doi: 10.1016/j.compedu.2019.04.002

[27] Aria M Cuccurullo C . bibliometrix: An R-tool for comprehensive science mapping analysis. J Informetrics. (2017) 11:959–75. doi: 10.1016/j.joi.2017.08.007

[28] Entezar-Almahdi E Mohammadi-Samani S Tayebi L Farjadian F . Recent advances in designing 5-fluorouracil delivery systems: A stepping stone in the safe treatment of colorectal cancer. Int J nanomedicine. (2020) 15:5445–58. doi: 10.2147/ijn.S257700 PMC7398750 32801699

[29] Mitchell MJ Billingsley MM Haley RM Wechsler ME Peppas NA Langer R . Engineering precision nanoparticles for drug delivery. Nat Rev Drug discovery. (2021) 20:101–24. doi: 10.1038/s41573-020-0090-8 PMC7717100 33277608

[30] Cabeza L Perazzoli G Mesas C Jiménez-Luna C Prados J Rama AR . Nanoparticles in colorectal cancer therapy: latest *in vivo* assays, clinical trials, and patents. AAPS PharmSciTech. (2020) 21:178. doi: 10.1208/s12249-020-01731-y 32591920

[31] Yao Y Zhou Y Liu L Xu Y Chen Q Wang Y . Nanoparticle-based drug delivery in cancer therapy and its role in overcoming drug resistance. Front Mol biosciences. (2020) 7:193. doi: 10.3389/fmolb.2020.00193 PMC7468194 32974385

[32] Rodríguez-Fanjul V Guerrero-López R Fernández-Varas B Perona R Sastre-Perona A Sastre L . Comparison of colorectal cancer stem cells and oxaliplatin-resistant cells unveils functional similarities. Cells. (2022) 11:511. doi: 10.3390/cells11030511 35159320 PMC8833894

[33] Das PK Islam F Lam AK . The roles of cancer stem cells and therapy resistance in colorectal carcinoma. Cells. (2020) 9:1392. doi: 10.3390/cells9061392 32503256 PMC7348976

[34] Lei X He Q Li Z Zou Q Xu P Yu H . Cancer stem cells in colorectal cancer and the association with chemotherapy resistance. Med Oncol. (2021) 38:43. doi: 10.1007/s12032-021-01488-9 33738588

[35] Xu XY Meng X Li S Gan RY Li Y Li HB . Bioactivity, health benefits, and related molecular mechanisms of curcumin: current progress, challenges, and perspectives. Nutrients. (2018) 10:1553. doi: 10.3390/nu10101553 30347782 PMC6213156

[36] Ning ST Lee SY Wei MF Peng CL Lin SY Tsai MH . Targeting colorectal cancer stem-like cells with anti-CD133 antibody-conjugated SN-38 nanoparticles. ACS Appl materials interfaces. (2016) 8:17793–804. doi: 10.1021/acsami.6b04403 27348241

[37] Akbari A Nazari-Khanamiri F Ahmadi M Shoaran M Rezaie J . Engineered exosomes for tumor-targeted drug delivery: A focus on genetic and chemical functionalization. Pharmaceutics. (2022) 15:66. doi: 10.3390/pharmaceutics15010066 36678695 PMC9865907

[38] Fu P Yin S Cheng H Xu W Jiang J . Engineered exosomes for drug delivery in cancer therapy: A promising approach and application. Curr Drug delivery. (2024) 21:817–27. doi: 10.2174/1567201820666230712103942 37438904

[39] Wu S Yun J Tang W Familiari G Relucenti M Wu J . Therapeutic m6A Eraser ALKBH5 mRNA-Loaded Exosome–Liposome Hybrid Nanoparticles Inhibit Progression of Colorectal Cancer in Preclinical Tumor Models. ACS Nano. (2023) 17:11838–54. doi: 10.1021/acsnano.3c03050 37310898

[40] Xiong L Wei Y Jia Q Chen J Chen T Yuan J . The application of extracellular vesicles in colorectal cancer metastasis and drug resistance: recent advances and trends. J nanobiotechnology. (2023) 21:143. doi: 10.1186/s12951-023-01888-1 37120534 PMC10148416

[41] Ye S You Q Song S Wang H Wang C Zhu L . Nanostructures and nanotechnologies for the detection of extracellular vesicle. Advanced Biol. (2023) 7:e2200201. doi: 10.1002/adbi.202200201 36394211

[42] Huang X O'Connor R Kwizera EA . Gold nanoparticle based platforms for circulating cancer marker detection. Nanotheranostics. (2017) 1:80–102. doi: 10.7150/ntno.18216 28217434 PMC5313055

[43] Wang N Chen L Huang W Gao Z Jin M . Current advances of nanomaterial-based oral drug delivery for colorectal cancer treatment. Nanomaterials (Basel). (2024) 14:557. doi: 10.3390/nano14070557 38607092 PMC11013305

[44] Tiwari A Saraf S Jain A Panda PK Verma A Jain SK . Basics to advances in nanotherapy of colorectal cancer. Drug delivery Trans Res. (2020) 10:319–38. doi: 10.1007/s13346-019-00680-9 31701486

[45] Abd Elhamid AS Heikal L Ghareeb DA Abdulmalek SA Mady O Teleb M . Engineering thermo/pH-responsive lactoferrin nanostructured microbeads for oral targeting of colorectal cancer. ACS Biomaterials Sci Engineering. (2024) 10:4985–5000. doi: 10.1021/acsbiomaterials.4c00666 39079030

[46] Ma Z Ma R Wang X Gao J Zheng Y Sun Z . Enzyme and PH responsive 5-flurouracil (5-FU) loaded hydrogels based on olsalazine derivatives for colon-specific drug delivery. Eur Polymer J. (2019) 118:64–70. doi: 10.1016/j.eurpolymj.2019.05.017

[47] London M Gallo E . Epidermal growth factor receptor (EGFR) involvement in epithelial-derived cancers and its current antibody-based immunotherapies. Cell Biol Int. (2020) 44:1267–82. doi: 10.1002/cbin.11340 32162758

[48] Udompornmongkol P Chiang BH . Curcumin-loaded polymeric nanoparticles for enhanced anti-colorectal cancer applications. J biomaterials applications. (2015) 30:537–46. doi: 10.1177/0885328215594479 26170212

[49] Danhier F Ansorena E Silva JM Coco R Le Breton A Préat V . PLGA-based nanoparticles: an overview of biomedical applications. J Controlled Release: Off J Controlled Release Society. (2012) 161:505–22. doi: 10.1016/j.jconrel.2012.01.043 22353619

[50] Shen J Choi S Qu W Wang Y Burgess DJ . *In vitro*-*in vivo* correlation of parenteral risperidone polymeric microspheres. J Controlled release: Off J Controlled Release Society. (2015) 218:2–12. doi: 10.1016/j.jconrel.2015.09.051 PMC4721561 26423236

[51] Yin T Wang L Yin L Zhou J Huo M . Co-delivery of hydrophobic paclitaxel and hydrophilic AURKA specific siRNA by redox-sensitive micelles for effective treatment of breast cancer. Biomaterials. (2015) 61:10–25. doi: 10.1016/j.biomaterials.2015.05.022 25996409

[52] Sharifi M Attar F Saboury AA Akhtari K Hooshmand N Hasan A . Plasmonic gold nanoparticles: Optical manipulation, imaging, drug delivery and therapy. J Controlled release: Off J Controlled Release Soc. (2019) 311-312:170–89. doi: 10.1016/j.jconrel.2019.08.032 31472191

[53] Ghorbani F Kokhaei P Ghorbani M Eslami M . Application of different nanoparticles in the diagnosis of colorectal cancer. Gene Rep. (2020) 328:1000–15. doi: 10.1016/j.genrep.2020.100896

[54] Pietro PD Strano G Zuccarello L Satriano C . Gold and silver nanoparticles for applications in theranostics. Curr topics medicinal Chem. (2016) 16:3069–102. doi: 10.2174/1568026616666160715163346 27426869

[55] Vines JB Yoon J-H Ryu N-E Lim D-J Park H . Gold nanoparticles for photothermal cancer therapy. Front Chem. (2019) 7:167. doi: 10.3389/fchem.2019.00167 31024882 PMC6460051

[56] Bruna T Maldonado-Bravo F Jara P Caro N . Silver nanoparticles and their antibacterial applications. Int J Mol Sci. (2021) 22:7202. doi: 10.3390/ijms22137202 34281254 PMC8268496

[57] MubarakAli D Thajuddin N Jeganathan K Gunasekaran M . Plant extract mediated synthesis of silver and gold nanoparticles and its antibacterial activity against clinically isolated pathogens. Colloids Surfaces B: Biointerfaces. (2011) 85:360–5. doi: 10.1016/j.colsurfb.2011.03.009 21466948

[58] Dahoumane SA Mechouet M Wijesekera K Filipe CDM Sicard C Bazylinski DA . Algae-mediated biosynthesis of inorganic nanomaterials as a promising route in nanobiotechnology – a review. Green Chem. (2017) 19:552–87. doi: 10.1039/C6GC02346K

[59] Rajeshkumar S Malarkodi C Vanaja M Gnanajobitha G Paulkumar K Gaurav K . Algae mediated green fabrication of silver nanoparticles and examination of its antifungal activity against clinical pathogens. Int J Metals. (2014) 1:1. doi: 10.1155/2014/692643

[60] Kalishwaralal K Deepak V Ramkumarpandian S Nellaiah H Sangiliyandi G . Extracellular biosynthesis of silver nanoparticles by the culture supernatant of Bacillus licheniformis. Materials Letters. (2008) 62:4411–3. doi: 10.1016/j.matlet.2008.06.051

[61] Petkova GA Záruba CK Zvátora P Král V . Gold and silver nanoparticles for biomolecule immobilization and enzymatic catalysis. Nanoscale Res Lett. (2012) 7:287. doi: 10.1186/1556-276x-7-287 22655978 PMC3447686

[62] Gholami-Shabani M Shams-Ghahfarokhi M Gholami-Shabani Z Akbarzadeh A Riazi G Ajdari S . Enzymatic synthesis of gold nanoparticles using sulfite reductase purified from Escherichia coli: A green eco-friendly approach. Process Biochem. (2015) 50:1076–85. doi: 10.1016/j.procbio.2015.04.004

[63] Iravani S . Green synthesis of metal nanoparticles using plants. Green Chem. (2011) 13:2638–50. doi: 10.1039/c1gc15386b

[64] Mittal AK Chisti Y Banerjee UC . Synthesis of metallic nanoparticles using plant extracts. Biotechnol advances. (2013) 31:346–56. doi: 10.1016/j.bioteChadv.2013.01.003 23318667

[65] González-Ballesteros N Prado-López S Rodríguez-González JB Lastra M Rodríguez-Argüelles. MC . Green synthesis of gold nanoparticles using brown algae Cystoseira baccata: Its activity in colon cancer cells. Colloids Surfaces B: Biointerfaces. (2017) 153:190–8. doi: 10.1016/j.colsurfb.2017.02.020 28242372

[66] Das P Fatehbasharzad P Colombo M Fiandra L Prosperi D . Multifunctional magnetic gold nanomaterials for cancer. Trends Biotechnol. (2019) 37:995–1010. doi: 10.1016/j.tibtech.2019.02.005 30862388

[67] Rajkumar S Prabaharan M . Theranostics based on iron oxide and gold nanoparticles for imaging- guided photothermal and photodynamic therapy of cancer. Curr topics medicinal Chem. (2017) 17:1858–71. doi: 10.2174/1568026617666161122120537 27875977

[68] Elsaesser A Howard CV . Toxicology of nanoparticles. Advanced Drug Del Rev. (2012) 64:129–37. doi: 10.1016/j.addr.2011.09.001 21925220

[69] Feliu N Docter D Heine M Del Pino P Ashraf S Kolosnjaj-Tabi J . *In vivo* degeneration and the fate of inorganic nanoparticles. Chem Soc Rev. (2016) 45:2440–57. doi: 10.1039/c5cs00699f 26862602

[70] Bardania H Jafari F Baneshi M Mahmoudi R Ardakani MT Safari F . Folic acid-functionalized albumin/graphene oxide nanocomposite to simultaneously deliver curcumin and 5-fluorouracil into human colorectal cancer cells: an *in vitro* study. BioMed Res Int. (2023) 2023:8334102. doi: 10.1155/2023/8334102 37304465 PMC10256446

[71] López-Méndez TB Sánchez-Álvarez M Trionfetti F Pedraz JL Tripodi M Cordani M . Nanomedicine for autophagy modulation in cancer therapy: a clinical perspective. Cell Biosci. (2023) 13:44. doi: 10.1186/s13578-023-00986-9 36871010 PMC9985235

[72] Shan X Li S Sun B Chen Q Sun J He Z . Ferroptosis-driven nanotherapeutics for cancer treatment. J Controlled release: Off J Controlled Release Society. (2020) 319:322–32. doi: 10.1016/j.jconrel.2020.01.008 31917296

[73] Kim SE Zhang L Ma K Riegman M Chen F Ingold I . Ultrasmall nanoparticles induce ferroptosis in nutrient-deprived cancer cells and suppress tumour growth. Nat nanotechnology. (2016) 11:977–85. doi: 10.1038/nnano.2016.164 PMC5108575 27668796

[74] Sharifi-Azad M Fathi M Cho WC Barzegari A Dadashi H Dadashpour M . Recent advances in targeted drug delivery systems for resistant colorectal cancer. Cancer Cell Int. (2022) 22:196. doi: 10.1186/s12935-022-02605-y 35590367 PMC9117978

[75] Zhong Y Su T Shi Q Feng Y Tao Z Huang Q . Co-administration of iRGD enhances tumor-targeted delivery and anti-tumor effects of paclitaxel-loaded PLGA nanoparticles for colorectal cancer treatment. Int J Nanomedicine. (2019) 14:8543–60. doi: 10.2147/ijn.S219820 PMC6830451 31802868

[76] Bai F Wang C Lu Q Zhao M Ban FQ Yu DH . Nanoparticle-mediated drug delivery to tumor neovasculature to combat P-gp expressing multidrug resistant cancer. Biomaterials. (2013) 34:6163–74. doi: 10.1016/j.biomaterials.2013.04.062 23706689

[77] Luis de Redín I Expósito F Agüeros M Collantes M Peñuelas I Allemandi D . *In vivo* efficacy of bevacizumab-loaded albumin nanoparticles in the treatment of colorectal cancer. Drug delivery Trans Res. (2020) 10:635–45. doi: 10.1007/s13346-020-00722-7 32040774

[78] Jing X Yang F Shao C Wei K Xie M Shen H . Role of hypoxia in cancer therapy by regulating the tumor microenvironment. Mol Cancer. (2019) 18:157. doi: 10.1186/s12943-019-1089-9 31711497 PMC6844052

[79] Chen J Jiang Z Xu W Sun T Zhuang X Ding J . Spatiotemporally targeted nanomedicine overcomes hypoxia-induced drug resistance of tumor cells after disrupting neovasculature. Nano Lett. (2020) 20:6191–8. doi: 10.1021/acs.nanolett.0c02515 32697585

[80] Nakatsu G Li X Zhou H Sheng J Wong SH Wu WK . Gut mucosal microbiome across stages of colorectal carcinogenesis. Nat Commun. (2015) 6:8727. doi: 10.1038/ncomms9727 26515465 PMC4640069

[81] Ghebretatios M Schaly S Prakash S . Nanoparticles in the food industry and their impact on human gut microbiome and diseases. Int J Mol Sci. (2021) 22:1942. doi: 10.3390/ijms22041942 33669290 PMC7920074

[82] Wang ZH Liu JM Li CY Wang D Lv H Lv SW . Bacterial biofilm bioinspired persistent luminescence nanoparticles with gut-oriented drug delivery for colorectal cancer imaging and chemotherapy. ACS Appl Materials Interfaces. (2019) 11:36409–19. doi: 10.1021/acsami.9b12853 31525949

[83] AL C d. SLO Schomann T de Geus-Oei LF Kapiteijn E Cruz LJ . Nanocarriers as a tool for the treatment of colorectal cancer. Pharmaceutics. (2021) 13:1321. doi: 10.3390/pharmaceutics13081321 34452282 PMC8399070

[84] Narayana S Gowda BHJ Hani U Shimu SS Paul K Das A . Inorganic nanoparticle-based treatment approaches for colorectal cancer: recent advancements and challenges. J Nanobiotechnol. (2024) 22:427. doi: 10.1186/s12951-024-02701-3 PMC11264527 39030546

[85] Kasi PB Mallela VR Ambrozkiewicz F Trailin A Liška V Hemminki K . Theranostics nanomedicine applications for colorectal cancer and metastasis: recent advances. Int J Mol Sci. (2023) 24:7922. doi: 10.3390/ijms24097922 37175627 PMC10178331

[86] Andoh V Ocansey DKW Naveed H Wang N Chen L Chen K . The advancing role of nanocomposites in cancer diagnosis and treatment. Int J Nanomedicine. (2024) 19:6099–126. doi: 10.2147/ijn.S471360 PMC11194004 38911500

[87] Rosenkrans ZT Ferreira CA Ni D Cai W . Internally responsive nanomaterials for activatable multimodal imaging of cancer. Adv Healthc Mater. (2021) 10:e2000690. doi: 10.1002/adhm.202000690 32691969 PMC7855763

[88] Liu LJ Ma QM Cao J Gao Y Han SC Liang Y . Recent progress of graphene oxide-based multifunctional nanomaterials for cancer treatment. Cancer Nanotechnology. (2021) 12:18. doi: 10.1186/s12645-021-00087-7

[89] Zhang Y Fang F Li L Zhang J . Self-assembled organic nanomaterials for drug delivery, bioimaging, and cancer therapy. ACS Biomater Sci Eng. (2020) 6:4816–33. doi: 10.1021/acsbiomaterials.0c00883 33455214

[90] Sun L Liu H Ye Y Lei Y Islam R Tan S . Smart nanoparticles for cancer therapy. Signal Transduct Target Ther. (2023) 8:418. doi: 10.1038/s41392-023-01642-x 37919282 PMC10622502

[91] Naeimi R Najafi R Molaei P Amini R Pecic S . Nanoparticles: The future of effective diagnosis and treatment of colorectal cancer? Eur J Pharmacol. (2022) 936:175350. doi: 10.1016/j.ejphar.2022.175350 36306928

[92] Ducreux M Bennouna J Adenis A Conroy T Lièvre A Portales F . Efficacy and safety of nab-paclitaxel in patients with previously treated metastatic colorectal cancer: a phase II COLO-001 trial. Cancer Chemother Pharmacol. (2017) 79:9–16. doi: 10.1007/s00280-016-3193-5 27866244

[93] Hamaguchi T Tsuji A Yamaguchi K Takeda K Uetake H Esaki T . A phase II study of NK012, a polymeric micelle formulation of SN-38, in unresectable, metastatic or recurrent colorectal cancer patients. Cancer Chemother Pharmacol. (2018) 82:1021–9. doi: 10.1007/s00280-018-3693-6 PMC6267673 30284603

[94] Rashidi N Davidson M Apostolopoulos V Nurgali K . Nanoparticles in cancer diagnosis and treatment: Progress, challenges, and opportunities. J Drug Delivery Sci Technology. (2024) 95:105599. doi: 10.1016/j.jddst.2024.105599

[95] Ji B Wei M Yang B . Recent advances in nanomedicines for photodynamic therapy (PDT)-driven cancer immunotherapy. Theranostics. (2022) 12:434–58. doi: 10.7150/thno.67300 PMC8690913 34987658

[96] Yang J Dai D Zhang X Teng L Ma L Yang YW . Multifunctional metal-organic framework (MOF)-based nanoplatforms for cancer therapy: from single to combination therapy. Theranostics. (2023) 13:295–323. doi: 10.7150/thno.80687 36593957 PMC9800740

[97] Iranpour S Bahrami AR Dayyani M Saljooghi AS Matin MM . A potent multifunctional ZIF-8 nanoplatform developed for colorectal cancer therapy by triple-delivery of chemo/radio/targeted therapy agents. J Materials Chem B. (2024) 12:1096–114. doi: 10.1039/d3tb02571c 38229578

