# Supplementary figures and images for: Mapping the intellectual structure and emerging trends on nanomaterials in colorectal cancer: a bibliometric analysis from 2003 to 2024
Source: Front Oncol. 2025 Jan 8;14:1514581. doi: 10.3389/fonc.2024.1514581 (PMC11750690; doi:10.3389/fonc.2024.1514581)

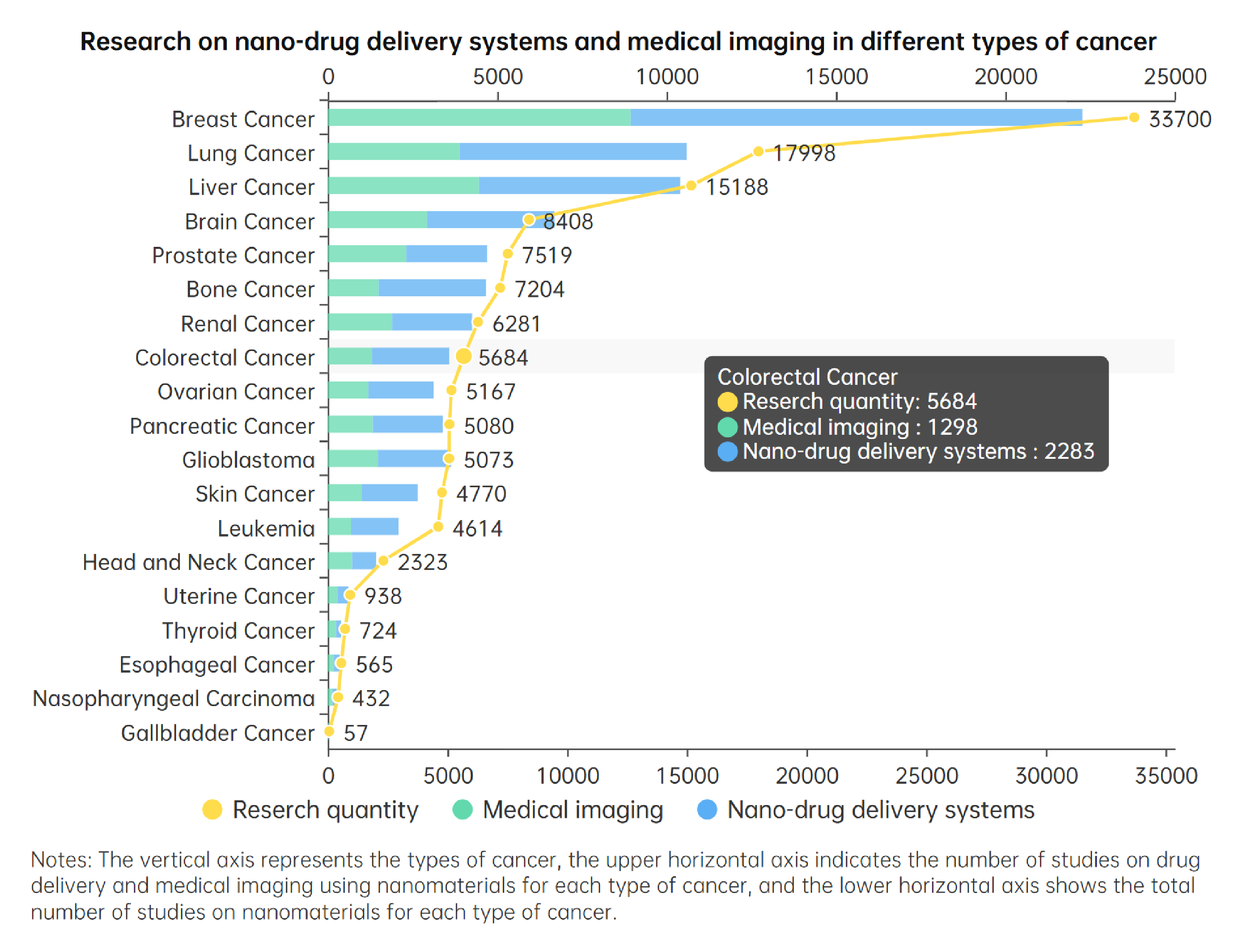

Supplement: Supplementary Figure 1 — Research on nano-drug delivery systems and medical imaging in different types of cancer. [file Image1.tif]

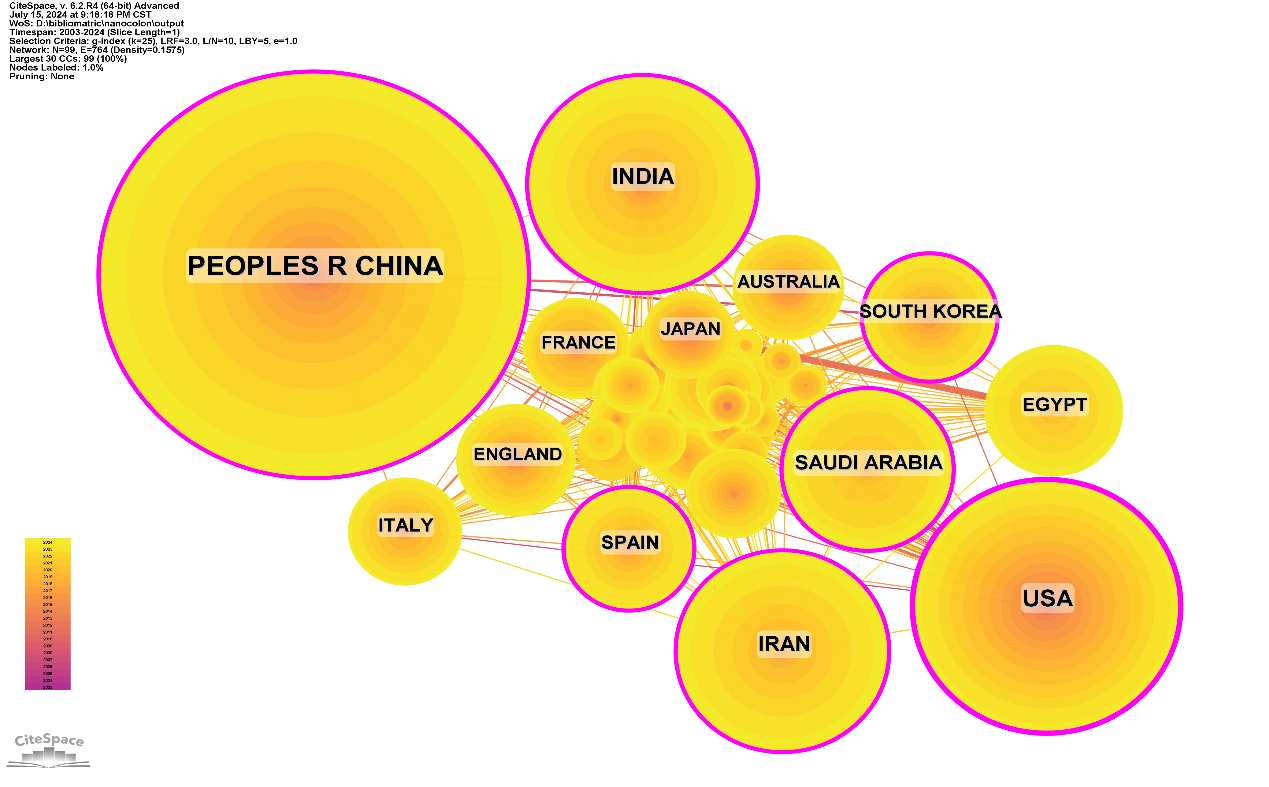

Supplement: Supplementary Figure 2 — Networks of country cooperation. [file Image2.tif]

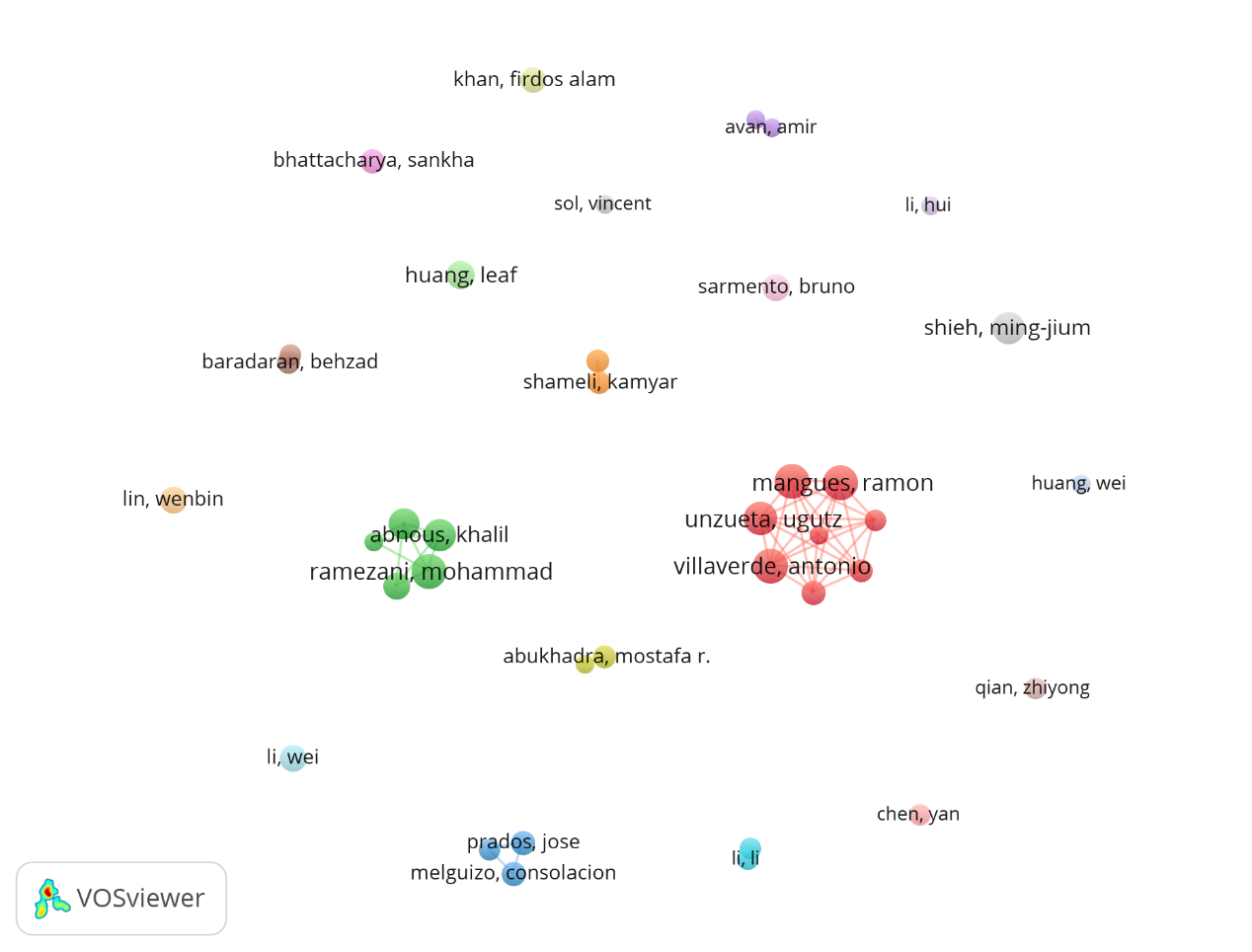

Supplement: Supplementary Figure 3 — Visual mapping of the authors and co-cited authors. (A) Networks of author cooperation; (B) Networks of co-cited authors. [file Image3.tif]

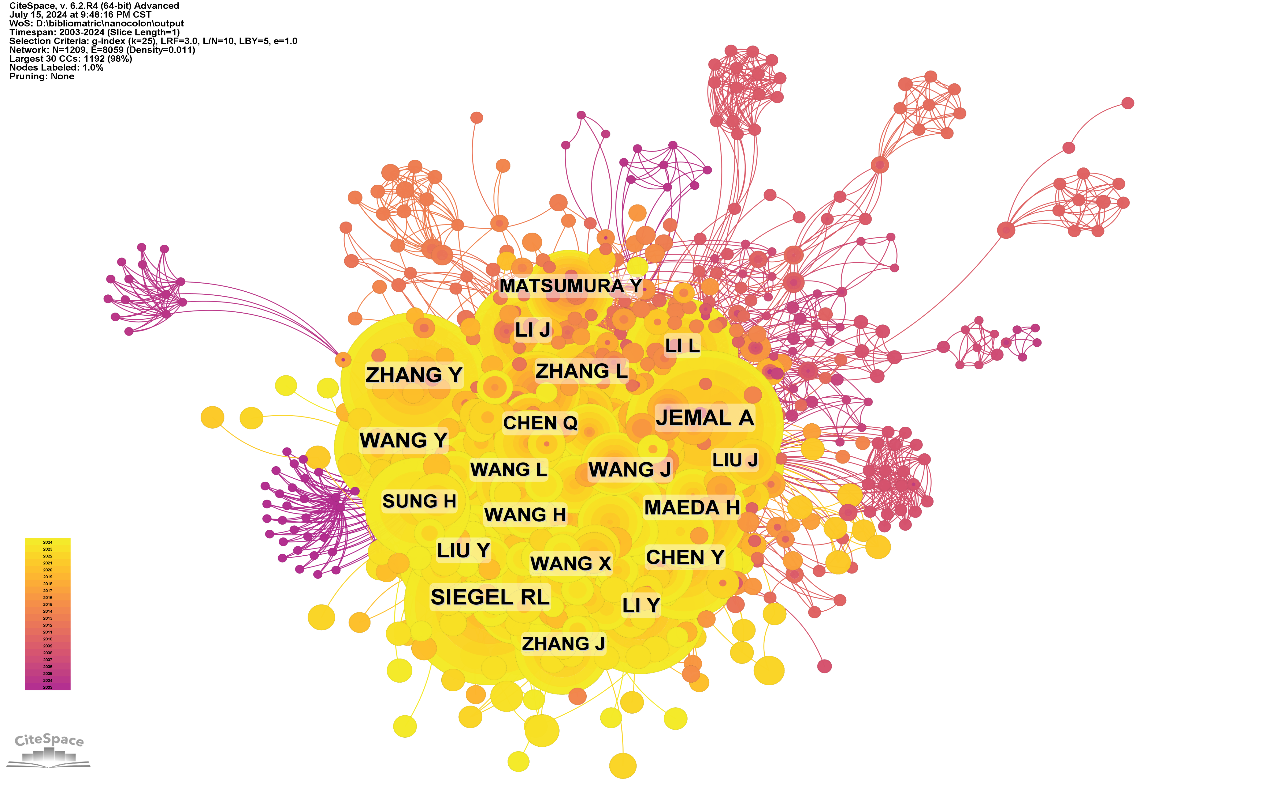

Supplement: Supplementary Figure 4 — Visual mapping of the journals and co-cited journals. (A) Density map of journal publications; (B) Networks of co-cited journals. [file Image4.tif]

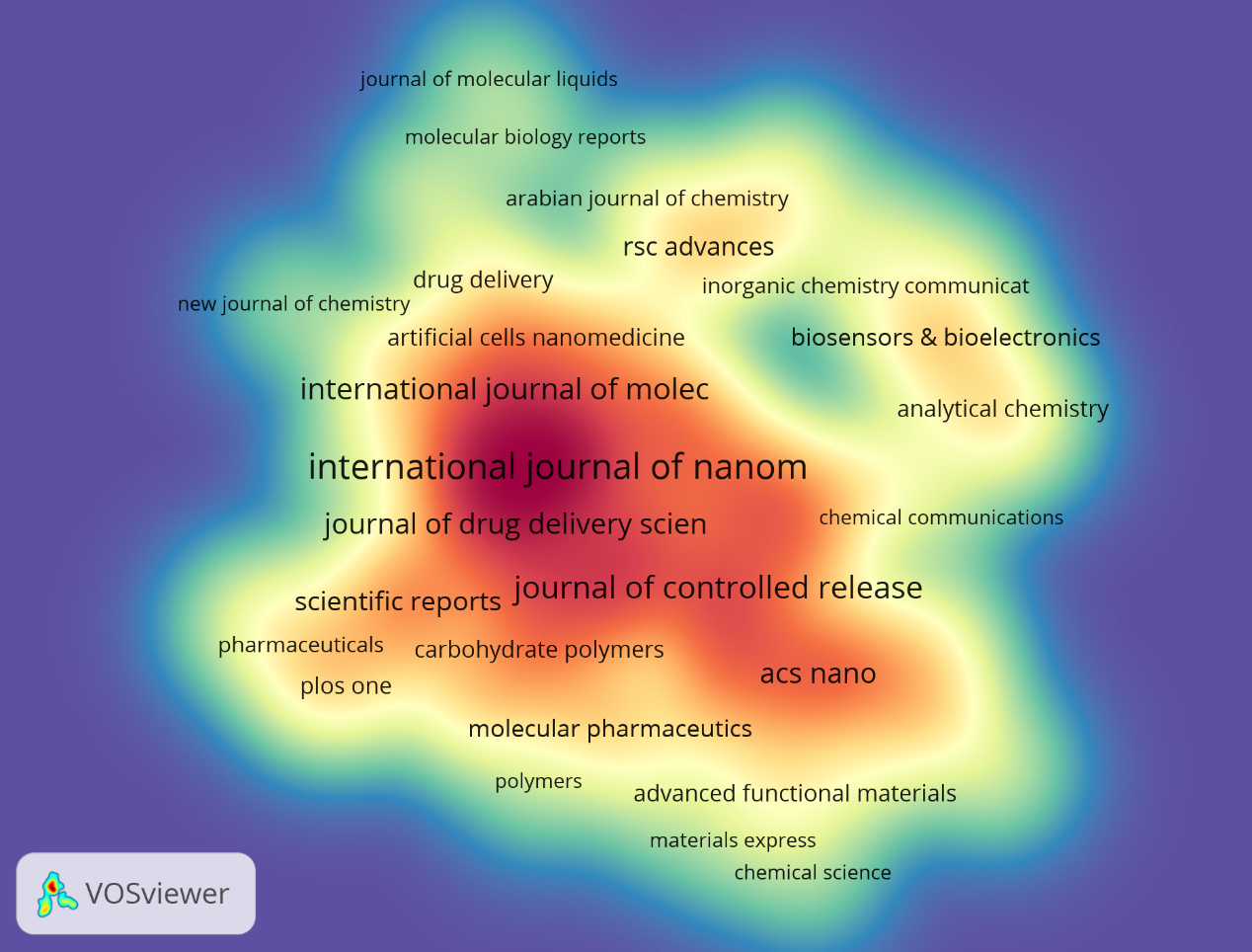

Supplement: Supplementary Figure 5 — Visual mapping of the co-cited references. (A) Networks of co-cited references; (B) Clustering of co-cited references; (C) Peak map of clustering of co-cited references over time. [file Image5.tif]

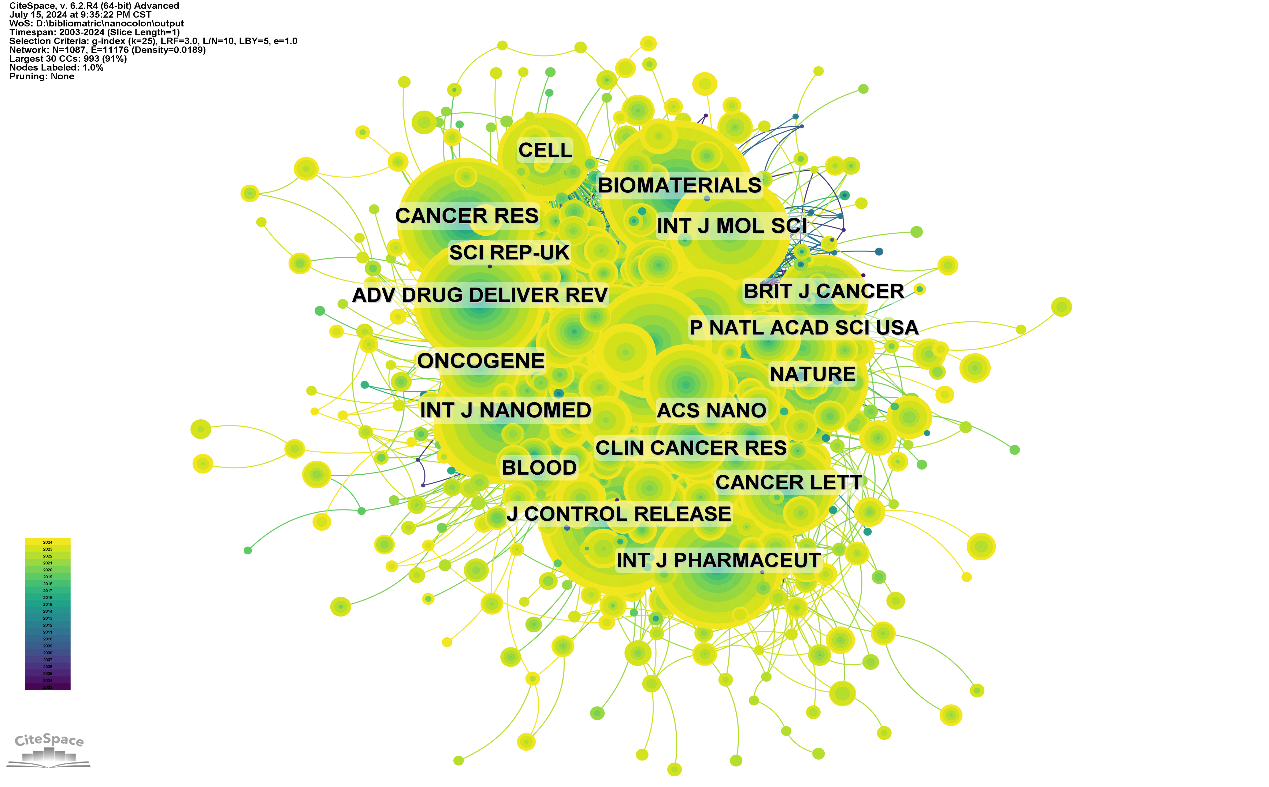

Supplement: Supplementary Figure 6 — Research trends in the field. (A) Keyword-based research trend quadrant chart (2003-2024); (B) Trend topics of the research field (2014 to 2024). [file Image6.tif]

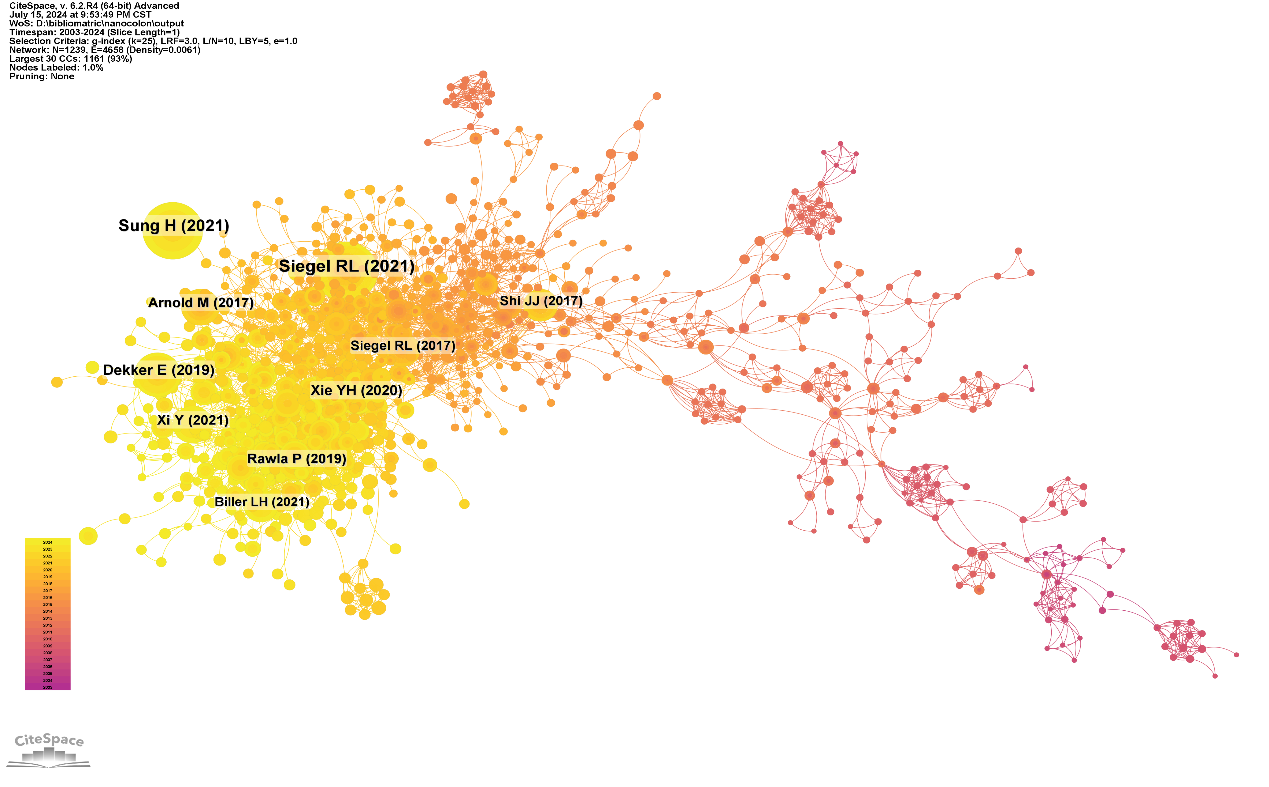

Supplement: Supplementary file 7 [file Image7.tif]

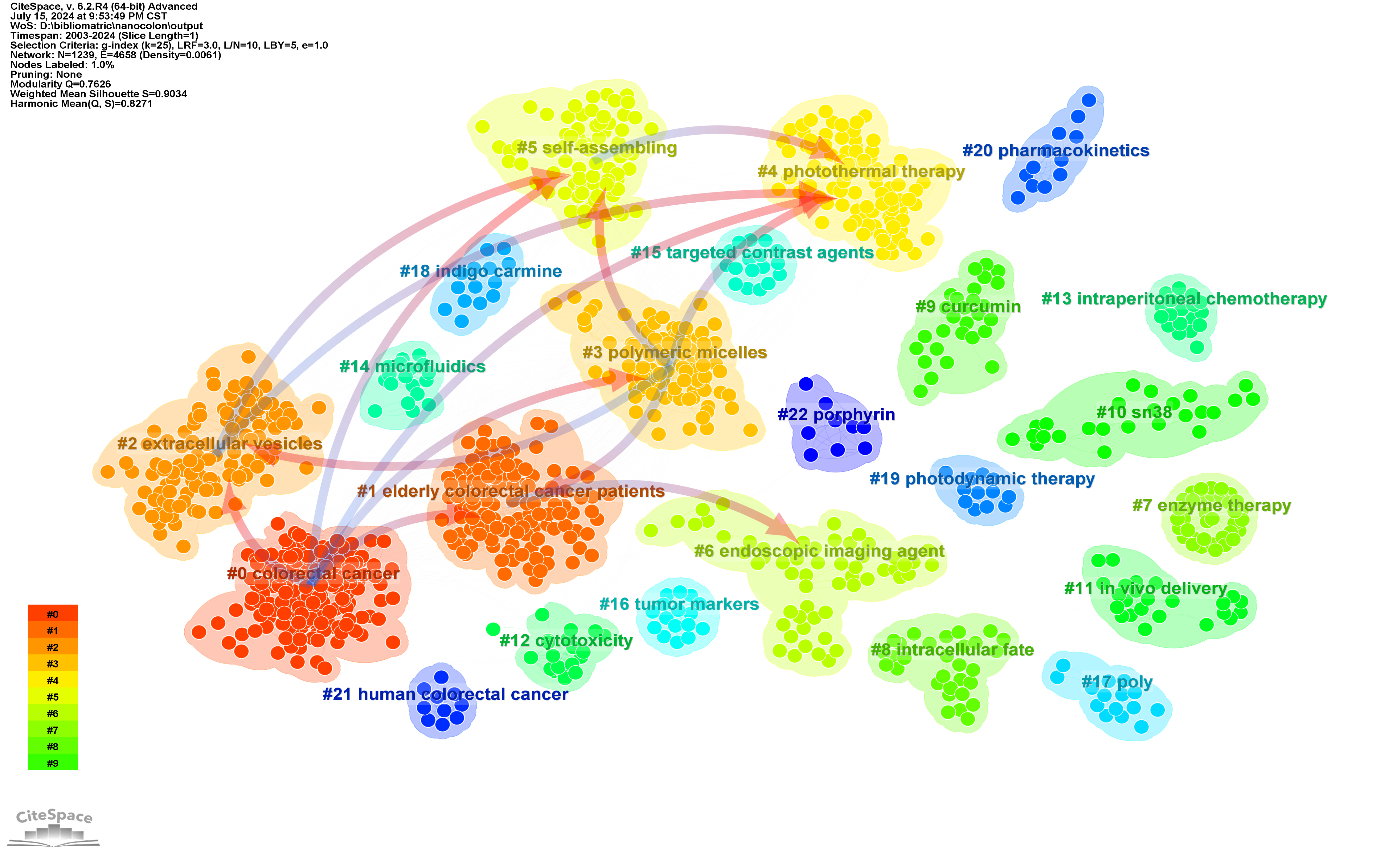

Supplement: Supplementary file 8 [file Image8.tif]

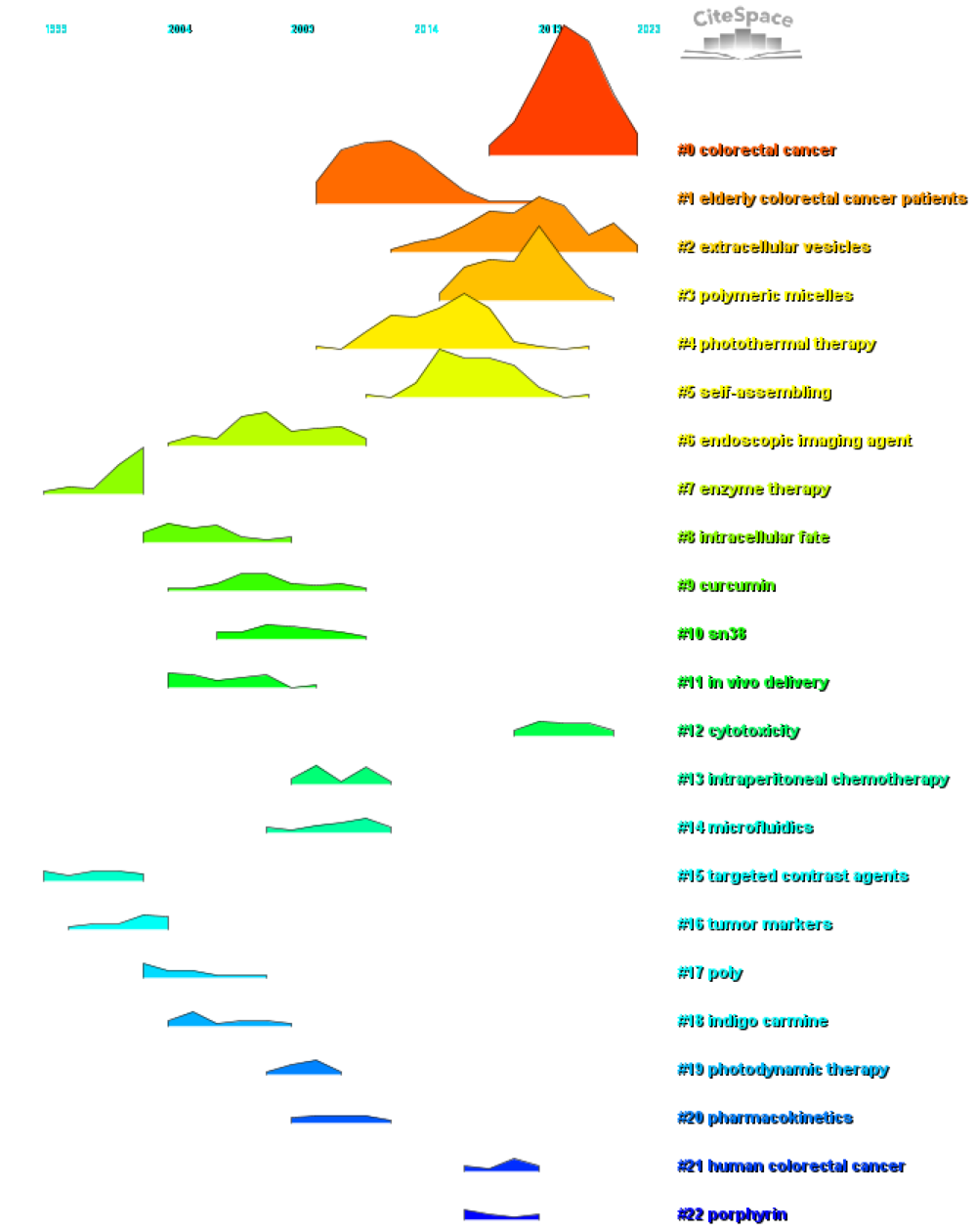

Supplement: Supplementary file 9 [file Image9.tif]

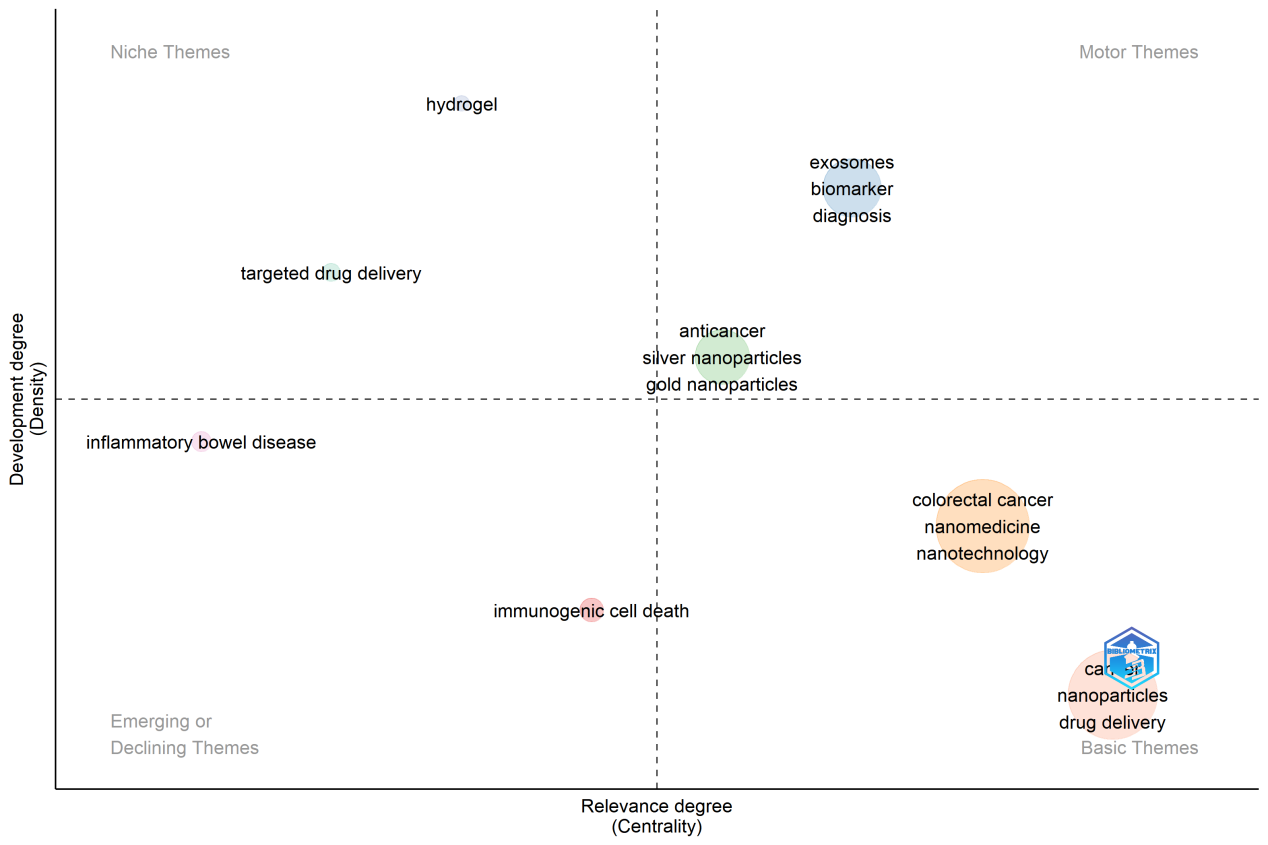

Supplement: Supplementary file 10 [file Image10.tif]

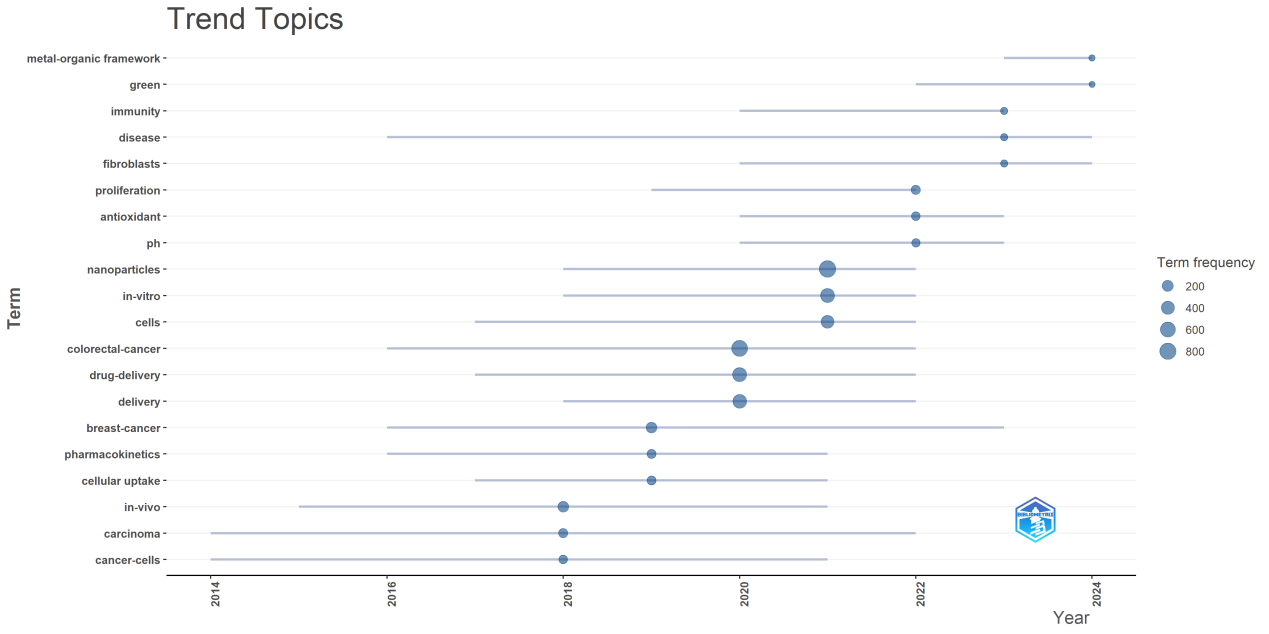

Supplement: Supplementary file 11 [file Image11.tif]
