# Supplementary material for: Mapping the intellectual structure and emerging trends on nanomaterials in colorectal cancer: a bibliometric analysis from 2003 to 2024
Source: Front Oncol. 2025 Jan 8;14:1514581. doi: 10.3389/fonc.2024.1514581 (PMC11750690; doi:10.3389/fonc.2024.1514581)
Supplement: Supplementary file 12 [file Table1.docx]

Supplementary Table S1. Top 10 productive countries/regions producing studies related to nanomaterials in CRC

| Rank | Country/Region | Counts | Percentage (%) | Total citations | Average citation | Centrality |
| --- | --- | --- | --- | --- | --- | --- |
| 1 | CHINA | 1349 | 36.63% | 39241 | 29.09 | 0.17 |
| 2 | USA | 556 | 15.10% | 25888 | 46.56 | 0.31 |
| 3 | INDIA | 397 | 10.78% | 8141 | 20.51 | 0.19 |
| 4 | IRAN | 334 | 9.07% | 6330 | 18.95 | 0.12 |
| 5 | SAUDI ARABIA | 227 | 6.16% | 4509 | 19.86 | 0.15 |
| 6 | EGYPT | 170 | 4.62% | 2841 | 16.71 | 0.08 |
| 7 | SOUTH KOREA | 158 | 4.29% | 4672 | 29.57 | 0.12 |
| 8 | SPAIN | 148 | 4.02% | 3475 | 23.48 | 0.14 |
| 9 | ITALY | 127 | 3.45% | 2982 | 23.48 | 0.03 |
| 10 | ENGLAND | 123 | 3.34% | 4974 | 40.44 | 0.08 |
