# Supplementary material for: Mapping the intellectual structure and emerging trends on nanomaterials in colorectal cancer: a bibliometric analysis from 2003 to 2024
Source: Front Oncol. 2025 Jan 8;14:1514581. doi: 10.3389/fonc.2024.1514581 (PMC11750690; doi:10.3389/fonc.2024.1514581)
Supplement: Supplementary file 13 [file Table2.docx]

Supplementary Table S2. Top 10 productive institutions producing studies related to nanomaterials in CRC

| Rank | Institution | Country | Counts | Total citations | Average citation |
| --- | --- | --- | --- | --- | --- |
| 1 | Chinese Academy of Sciences | CHINA | 159 | 6310 | 39.69 |
| 2 | Egyptian Knowledge Bank | EGYPT | 159 | 2763 | 17.38 |
| 3 | Mashhad University Medical Science | IRAN | 75 | 1624 | 21.65 |
| 4 | King Saud University | SAUDI ARABIA | 65 | 1368 | 21.05 |
| 5 | Zhejiang University | CHINA | 63 | 1502 | 23.84 |
| 6 | Islamic Azad University | IRAN | 60 | 778 | 12.97 |
| 7 | Sun Yat-Sen University | CHINA | 58 | 1702 | 29.34 |
| 8 | Fudan University | CHINA | 57 | 1809 | 31.74 |
| 9 | Jilin University | CHINA | 57 | 1704 | 29.89 |
| 10 | Sichuan University | CHINA | 56 | 2019 | 36.05 |
