# Supplementary material for: Mapping the intellectual structure and emerging trends on nanomaterials in colorectal cancer: a bibliometric analysis from 2003 to 2024
Source: Front Oncol. 2025 Jan 8;14:1514581. doi: 10.3389/fonc.2024.1514581 (PMC11750690; doi:10.3389/fonc.2024.1514581)
Supplement: Supplementary file 14 [file Table3.docx]

Supplementary Table S3. Top 10 co-citation references related to nanomaterials in CRC

| Rank | Co-cited reference | Journal | Author(s) | Total citations | IF(2023) |
| --- | --- | --- | --- | --- | --- |
| 1 | Cancer Statistics, 2021 | CA-A CANCER JOURNAL FOR CLINICIANS | Siegel RL et al. | 178 | Q1/503.1 |
| 2 | Colorectal cancer | LANCET | Dekker E et al. | 87 | Q1/98.4 |
| 3 | Comprehensive review of targeted therapy for colorectal cancer | SIGNAL TRANSDUCTION AND TARGETED THERAPY | Xie YH et al. | 68 | Q1/40.8 |
| 4 | Global colorectal cancer burden in 2020 and projections to 2040 | TRANSLATIONAL ONCOLOGY | Xi Y et al. | 63 | Q1/4.5 |
| 5 | Global patterns and trends in colorectal cancer incidence and mortality | GUT | Arnold M et al. | 61 | Q1/23 |
| 6 | Epidemiology of colorectal cancer: incidence, mortality, survival, and risk factors | PRZEGLAD GASTROENTEROLOGICZNY | Rawla P et al. | 57 | Q3/1.7 |
| 7 | Cancer nanomedicine: progress, challenges and opportunities | NATURE REVIEWS CANCER | Shi JJ et al. | 51 | Q1/72.5 |
| 8 | Diagnosis and Treatment of Metastatic Colorectal Cancer: A Review | JAMA-JOURNAL OF THE AMERICAN MEDICAL ASSOCIATION | Biller LH et al. | 46 | Q1/63.1 |
| 9 | Controlled drug delivery vehicles for cancer treatment and their performance | SIGNAL TRANSDUCTION AND TARGETED THERAPY | Senapati S et al. | 39 | Q1/40.8 |
| 10 | Nano based drug delivery systems: recent developments and future prospects | JOURNAL OF NANOBIOTECHNOLOGY | Patra JK et al. | 38 | Q1/10.6 |
