# Supplementary material for: Mapping the intellectual structure and emerging trends on nanomaterials in colorectal cancer: a bibliometric analysis from 2003 to 2024
Source: Front Oncol. 2025 Jan 8;14:1514581. doi: 10.3389/fonc.2024.1514581 (PMC11750690; doi:10.3389/fonc.2024.1514581)
Supplement: Supplementary file 15 [file Table4.docx]

Supplementary Table S4. Nanomaterials in clinical trials for CRC.

| **Particle type** | **Product** | **NCT Number** | **Study Title** | **Indication** | **Study Status** | **Phases** | **Refs.** |
| --- | --- | --- | --- | --- | --- | --- | --- |
| Liposome | | | | | | | |
|  | PROMITIL | NCT01705002 | Intravenously administered Pegylated Liposomal Mitomycin-C Lipid-based Prodrug (PROMITIL) in cancer patients with solid tumors. | Metastatic Colorectal Cancer | Completed | PHASE Ⅰ | 30,90 |
|  | Aroplatin | NCT00043199 | A safety and effectiveness study of Aroplatin in patients with advanced colorectal cancer resistant to standard therapies | Colorectal Cancer | Unknown | PHASE Ⅱ | 30 |
|  | SN-38 liposome | NCT00311610 | Liposomal SN-38 in treating patients with metastatic colorectal cancer | Colorectal Cancer | Completed | PHASE Ⅱ | 30 |
|  | CPX-1 liposome | NCT00361842 | Multicenter study of CPX-1 (Irinotecan HCl: Floxuridine) liposome injection in patients with advanced colorectal cancer | Colorectal Cancer | Completed | PHASE Ⅱ | 30 |
|  | MM-398 | NCT02640365 | A dose escalation study of MM-398 plus irinotecan in patients with unresectable advanced cancer | Unresectable Advanced Cancer | Completed | PHASE Ⅰ | 30 |
|  | NAL-IRI | NCT03368963 | TAS102 in combination with NAL-IRI in advanced GI cancers | Colorectal Adenocarcinoma,  Gastric Adenocarcinoma,  Metastatic Pancreatic Adenocarcinoma,  Non-Resectable Cholangiocarcinoma | Recruiting | PHASE Ⅰ, Ⅱ | 30 |
|  | Nal-IRI | NCT03337087 | Liposomal irinotecan, fluorouracil, leucovorin calcium, and rucaparib in treating patients with metastatic pancreatic, colorectal, gastroesophageal, or biliary cancer | Metastatic Colorectal Cancer | Active, not recruiting | PHASE Ⅰ, Ⅱ | 30,91 |
|  | NAL-IRI | NCT06643793 | Evaluation of Irinotecan Liposome (II) combined with 5-FU, LV, and bevacizumab for mCRC | Colorectal Cancer | Not yet recruiting | PHASE Ⅱ | ClinicalTrials.gov |
|  | NAL-IRI | NCT06341296 | Phase II study of Irinotecan Liposomes in first-line treatment of metastatic colorectal cancer | Metastatic Colorectal Cancer | Recruiting | PHASE Ⅱ | ClinicalTrials.gov |
|  | NAL-IRI | NCT05969899 | Liposomal Irinotecan based FOLFIRI with bevacizumab in first-line treatment of advanced colorectal cancer | Colorectal Cancer | Active, not recruiting | PHASE Ⅱ | ClinicalTrials.gov |
| Lipid nanoparticles | | | | | | | |
|  | TKM-080301 | NCT01437007 | TKM-080301 for primary or secondary liver cancer | Metastatic Colorectal Cancer | Completed | PHASE Ⅰ | 30,91 |
| Albumin Nanoparticles | | | | | | | |
|  | Abraxane | NCT02103062 | Phase 2 study with Abraxane (Nab®Paclitaxel) in metastatic colorectal cancer | Colorectal Cancer | Completed | PHASE Ⅱ | 92 |
|  | Abraxane | NCT01730586 | Abraxane in CIMP-High colorectal and small bowel adenocarcinomas | Colorectal Cancer,  Gastric Cancer | Completed | PHASE Ⅱ | ClinicalTrials.gov |
| polymeric nanoparticles | | | | | | | |
|  | Polymeric NPs | NCT03774680 | Targeted polymeric nanoparticles loaded with cetuximab and decorated with somatostatin analogue to colon cancer | Colorectal Cancer | Unknown | PHASE Ⅰ | 30,91 |
| polymeric micelle | | | | | | | |
|  | NK012 | NCT01238939 | Study of NK012 and 5-FU/LV in solid tumors followed by dose expansion in colorectal cancer | Metastatic Colorectal Cancer | Completed | PHASE Ⅰ | 93 |
| PEGylated protein | | | | | | | |
|  | PEG-rhG-CSF | NCT02805166 | PEG-rhG-CSF in patients with malignant solid tumors receiving chemotherapy | Malignant Solid Tumors | Completed | PHASE Ⅳ | 30 |
| Carbon Nanoparticles | | | | | | | |
|  | Carbon NPs | NCT03350945 | Application of carbon nanoparticles in laparoscopic colorectal surgery | Colorectal Cancer | Unknown | NA | 30,94 |
|  | CNSI-Fe(II) | NCT06048367 | Carbon Nanoparticle-Loaded Iron [CNSI-Fe(II)] in the treatment of advanced solid tumor | Colorectal Cancer,  Advanced Solid Tumor,  Lung Cancer,  Pancreas Cancer,  Breast Cancer,  Thyroid Cancer,  Cervical Cancer,  Ovarian Cancer,  Vulva Cancer | Recruiting | PHASE Ⅰ | ClinicalTrials.gov |
| Silica Nanoparticles | | | | | | | |
|  | Silica Nanoparticles | NCT02106598 | Targeted silica nanoparticles for real-time image-guided intraoperative mapping of nodal metastases | Metastatic Colorectal Cancer | Active, not recruiting | PHASE Ⅰ, Ⅱ | 30,91,94 |
| Gadolinium Nanoparticles | | | | | | | |
|  | AGuIX | NCT04899908 | Stereotactic brain-directed radiation with or without Aguix Gadolinium-Based Nanoparticles in brain metastases | Colorectal Cancer,  Brain Cancer,  Brain Metastases,  Melanoma,  Lung Cancer,  Breast Cance,  HER2-positive Breast Cancer,  Gastrointestinal Cancer | Recruiting | PHASE Ⅱ | ClinicalTrials.gov |
